# A general chemical crosslinking strategy for structural analyses of weakly interacting proteins applied to preTCR–pMHC complexes

**DOI:** 10.1016/j.jbc.2021.100255

**Published:** 2021-01-08

**Authors:** Réka Mizsei, Xiaolong Li, Wan-Na Chen, Monika Szabo, Jia-Huai Wang, Gerhard Wagner, Ellis L. Reinherz, Robert J. Mallis

**Affiliations:** 1 Laboratory of Immunobiology, Dana Farber Cancer Institute, Boston, Massachusetts, USA; 2 Department of Biochemistry, Semmelweis University, Budapest, Hungary; 3 Department of Medical Oncology, Dana Farber Cancer Institute, Boston, Massachusetts, USA; 4 Department of Medicine, Harvard Medical School, Boston, Massachusetts, USA; 5 Department of Biological Chemistry and Molecular Pharmacology, Harvard Medical School, Boston, Massachusetts, USA; 6 Monash Institute of Pharmaceutical Sciences, Monash University, Parkville, VIC, Australia; 7 Department of Cancer Biology, Dana Farber Cancer Institute, Boston, Massachusetts, USA; 8 Department of Pediatrics, Harvard Medical School, Boston, Massachusetts, USA; 9 Department of Dermatology, Harvard Medical School, Boston, Massachusetts, USA

**Keywords:** nuclear magnetic resonance (NMR), protein–protein interaction, immunology, molecular modeling, T-cell receptor (TCR), pre-T cell receptor (preTCR), peptide–major histocompatibility complex molecule (pMHC), pseudocontact shift (PCS) NMR, thymocyte development, homobifunctional crosslinking, NMR, nuclear magnetic resonance, PCS, pseudocontact chemical shift, pMHC, peptide–major histocompatibility complex, SEC, size-exclusion chromatography, TCR, T-cell receptor

## Abstract

T lymphocytes discriminate between healthy and infected or cancerous cells *via* T-cell receptor-mediated recognition of peptides bound and presented by cell-surface-expressed major histocompatibility complex molecules (MHCs). Pre-T-cell receptors (preTCRs) on thymocytes foster development of αβT lymphocytes through their β chain interaction with MHC displaying self-peptides on thymic epithelia. The specific binding of a preTCR with a peptide–MHC complex (pMHC) has been identified previously as forming a weak affinity complex with a distinct interface from that of mature αβTCR. However, a lack of appropriate tools has limited prior efforts to investigate this unique interface. Here we designed a small-scale linkage screening protocol using bismaleimide linkers for determining residue-specific distance constraints between transiently interacting protein pairs in solution. Employing linkage distance restraint-guided molecular modeling, we report the oriented solution docking geometry of a preTCRβ–pMHC interaction. The linkage model of preTCRβ–pMHC complex was independently verified with paramagnetic pseudocontact chemical shift (PCS) NMR of the unlinked protein mixtures. Using linkage screens, we show that the preTCR binds with differing affinities to peptides presented by MHC in solution. Moreover, the C-terminal peptide segment is a key determinant in preTCR–pMHC recognition. We also describe the process for future large-scale production and purification of the linked constructs for NMR, X-ray crystallography, and single-molecule electron microscopy studies.

Adaptive T-cell-mediated immunity is driven by activation of T cells *via* their surface T-cell receptors (TCRs) (1, 2, 3, 4). αβT cells arise from T-lineage progenitors in the thymus that have been subjected to a series of selection events at discrete stages of intrathymic development, preserving useful specificities while eliminating harmful ones (5, 6, 7, 8). This thymic education creates a functional T-cell repertoire incorporating TCRs capable of recognizing myriad antigenic peptide fragments presented by major histocompatibility complex molecules (pMHC) on antigen presenting cell surfaces (9, 10, 11, 12, 13, 14).

The maturation steps of thymocytes are identified by expression of cell surface markers [reviewed in (5, 6, 7, 8)]. Early thymocytes lack coreceptors CD4 and CD8 and are therefore termed double-negative (DN) cells. DN development is further divided into four stages based on expression of CD44 and CD25 markers. In the DN3 stage (CD44^−^, CD25^+^) TCRβ gene rearrangements occur and the thymocytes undergo beta-selection, which requires that TCRβ chains are produced and capable of pairing with a surrogate α chain, termed pTα, to generate the diversity of preTCRs. Each thymocyte within the αβT-cell lineage can be activated through a preTCR whose clone-specific pTα-β heterodimer is assembled with the same signal-initiating invariant CD3 subunits (CD3εγ, CD3εδ, and CD3ζζ) as on the cell surface of mature thymocytes and peripheral T cells (5, 15). A functional preTCR signaling platform allows for the cellular developmental transition beyond the beta-selection checkpoint by upregulating the expression of CD4 and CD8, to generate double-positive (DP) thymocytes. DP cells rearrange their α chain loci, activating transcription, and translation of the TCRα subunit for assembly with the other TCR components to produce the mature αβTCR. The αβTCR is a membrane-bound multiprotein complex, which is composed of an antigen-binding disulfide-linked αβ heterodimer that noncovalently associates with the CD3 subunits [(15) and references therein]. TCRα and β subunits form a variable VαVβ module, which binds pMHC, and a CαCβ constant region module, which interacts with the CD3 ectodomains (1, 2, 4, 5, 16). The VαVβ antigen recognition module utilizes six complementarity determining region (CDR) loops to recognize pMHC. Three CDRα and three CDRβ loops directly interact with the antigen presenting α1 and α2 domains of the MHC along with the peptide displayed (1, 4, 15, 17).

The structural features of the preTCR heterodimer are similar to the mature TCRαβ heterodimer, since they use the same β subunit. However, in the preTCR β is paired with an invariant pTα, therefore lacking the Vα domain of the mature TCRα and exposing novel interaction surfaces on Vβ. Recent studies on purified proteins showed that preTCR binds pMHC, and the ligation fostered elevated levels of calcium influx in thymocytes, indicative of active signaling (18). The selective proliferation of thymocytes whose preTCR can bind self-pMHC also suggested that the β repertoire could be skewed prior to TCRα rearrangements and canonical TCRαβ positive and negative selection. Chemical shift perturbation and cross-saturation transfer NMR studies mapped the interacting residues of N15β and N30β and showed that the β subunit of preTCR uses not only CDR regions as does the mature TCR, but also the distinctive Vβ patch for ligand binding (18, 19, 20). Mutational analysis independently confirmed the importance of patch residues for functional ligand binding interactions (18, 21). The Vβ patch is available for pMHC only in the preTCR, since it is occluded by the Vα domain of the TCRα subunit, when it replaces pTα upon αβTCR formation. For the Vβ patch to serve as a recognition element in the preTCR implies that the β chain docking to pMHC is distinct in the preTCR *versus* in the αβTCR, but the precise orientation of the preTCR–pMHC interaction is still unknown, with several docking modes feasible (20). Because this interaction is notably different from the “CDR-only” binding mode utilized by the mature αβTCR, including that of the N15 αβTCR (17), it is of the utmost importance to narrow the possible interplay modes to achieve atomic level resolution structural information on the interaction sites.

The interaction between N15β and VSV8/K^b^ was previously determined to have a K_D_ = 400 μM by NMR titration (20), an affinity that is out of the sensitive detection range of the majority of protein affinity determination methods (22). Due to the weak affinity, attempts to obtain distance restraints by NMR or to cocrystallize the complex have been unsuccessful. We thus sought to develop a methodology to characterize the solution docking geometry between β and pMHC as a proxy for preTCR–pMHC interaction. Using bismaleimide functionalized polyethylene glycol and other flexible linkers, we were able to generate a system for linking the low-affinity β-pMHC complex in a manner that promotes the appropriate pairing without fostering nonspecific interactions. This method will prove useful both in generating substrates for structural studies and in characterizing weak interactions directly.

## Results

### Generation of unimolecular preTCRβ–pMHC complexes *via* chemical linkage

**Figure 1:**
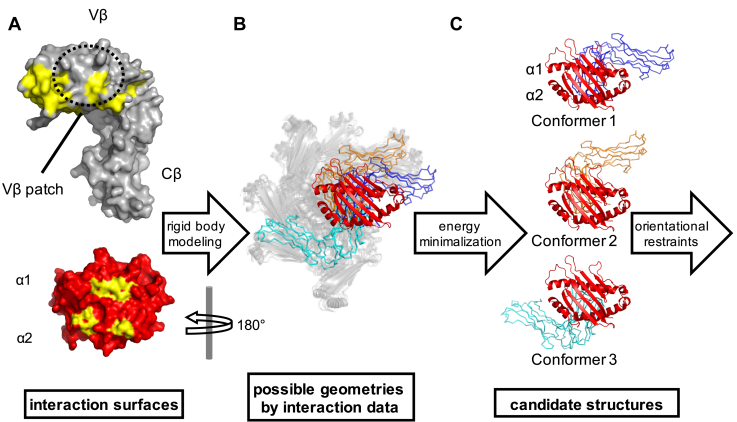
**Features of the preTCR-pMHC interaction.***A*, high-resolution crystal structures of individual proteins N15β and VSV8/K^b^ were determined previously (PDB: 3Q5Y, 1KPU), with K^b^ shown truncated at residue P185 [(K^b^-t), (20)]. The contact residues between N15β and VSV8/K^b^ (colored *yellow*) were identified by NMR cross-saturation (18) or chemical shift changes (20) using an unlabeled VSV8 peptide. The interaction surfaces of N15Vβ include the complementarity-determining regions (CDRs) as well as an exposed hydrophobic Vβ patch characteristic only of the preTCR. The binding interface of K^b^-t localized to the peptide binding groove and the framing α1- and α2 helices. K^b^-t was rotated 180° about the y axis in panels *B* and *C*. *B*, the NMR interaction map could be satisfied by models with wide angular distribution of N15β relative to VSV8/K^b^-t, which is represented by HADDOCK (48) modeled complexes prior to selection of low energy clusters. The three sets of binding modes highlighted after energy minimization in Panel C are noted by colorization. *C*, the three lowest energy binding conformations from Ref. (20) utilize two distinct docking orientations, which could not be resolved due to lack of experimental orientational restraints. MHC α1 and α2 helices are labeled to show the orientation of the complexes.

Although residues participating in the interaction between N15β and VSV8/K^b^ were identified by NMR cross-saturation or chemical shift perturbation (Fig. 1*A*), the docking orientation of the complex could not be unambiguously resolved from the experimental data. Figure 1*B* shows a wide angular distribution of the preTCR–pMHC models that use the interaction surface determined by NMR previously (18, 20) without regard for surface interaction parameters. Molecular modeling produced three low energy clusters with binding conformations that utilize two distinct docking orientations (20), which could not be resolved due to lack of directional restraints (Fig. 1*C*). To this end, we developed a chemical linkage strategy to investigate further the binding mode of the N15β-VSV8/K^b^-t2 complex as a single molecule. K^b^-t2 is a truncated version of K^b^ (derived from K^b^-t in Ref (20)), which consists of the antigen presenting α1 and α2 domains of the pMHC but lacks the α3 domain and the invariant β2 microglobulin subunit (β2M). K^b^-t2 was adapted for linkage by mutating Cys121 to Gln to retain only the native disulfide pairing Cys101 and Cys164 that is important for structural integrity. VSV8/K^b^-t has been shown to interact with a similar surface of N15β as VSV8/H-2K^b^/β2M (20).

**Figure 2:**
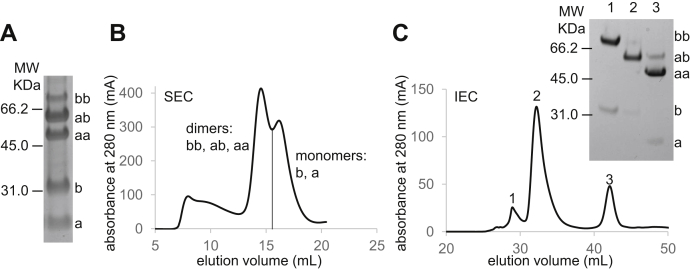
**Characterization and purification of products of the bifunctional linkage reaction.***A*, the components of the 1,11-bis(maleimido)triethylene glycol (BMPEG3) linkage reaction between K^b^-t2 K68C and N15β S30C were visualized by Coomassie blue staining after separation by reducing sodium dodecyl sulfate–polyacrylamide gel electrophoresis (SDS-PAGE). The symbols a and b label the monomeric K^b^-t2 K68C (22.3 kDa) and N15β S30C (27.4 kDa) respectively; the corresponding BMPEG3 linked dimers are represented by aa, ab, and bb. Positions of 66.2, 45.0, and 31.0 kDa molecular weight standards are shown. *B*, the dimers (aa, ab, bb) were separated from the monomeric forms (a, b) by size-exclusion chromatography (SEC) as described in Experimental procedures. *C*, the dimer-containing fractions from SEC were separated by ion exchange chromatography (IEC). Composition of the three major IEC peaks were verified by SDS-PAGE (*inset* of *C*). After concentration and buffer exchange, protein from IEC peak 2 (lane 2) was used for NMR (Fig. 6*B*).

We then generated single Cys mutants of N15β and VSV8/K^b^-t2 for pairwise linkage proximal to the interaction surface. N15β and VSV8/K^b^-t2 variants were mixed in solution with bismaleimide crosslinkers (Fig. S1) that conjugate between sulfhydryl groups. The species in the reaction mixture were identified by their apparent molecular weight using sodium dodecyl sulfate–polyacrylamide gel electrophoresis (SDS-PAGE). Figure 2*A* demonstrates that the 1,11-bis(maleimido)triethylene glycol (BMPEG3) reaction of N15β S30C and VSV8/K^b^-t2 K68C yielded crosslinked N15β and VSV8/K^b^-t2 heterodimers (ab) as well as (N15β)_2_ and (VSV8/K^b^-t2)_2_ homodimers (bb and aa, respectively), while a subset of molecules remained as N15β and VSV8/K^b^-t2 monomers (b and a). In subsequent figures only the region of the gel containing the dimeric products is focused on to illustrate the relative yields of each dimer. Afterward, heterodimers (ab) were purified in two chromatographic steps. The dimeric components could be separated from the monomers by size-exclusion chromatography (SEC), but the individual dimers could not be resolved (Fig. 2*B*). Since the theoretical isoelectric points (pI) (https://www.expasy.org/compute_pi/, accessed July 14, 2020) of N15β (pI = 6.76) and VSV8/K^b^-t2 (pI = 4.97) are significantly different, we were able to purify the “ab” heterodimers using anion exchange chromatography for use in NMR spectroscopy (see below). We used two successive rounds of SEC to generate samples for crystallographic screening as linkage reactions *per se* yielding mainly “ab” heterodimers were selected for crystallization (described below).

### Bifunctional linkers react with mixtures of single Cys mutant protein pairs to produce homodimers and heterodimers with structurally indicative distributions

**Figure 3:**
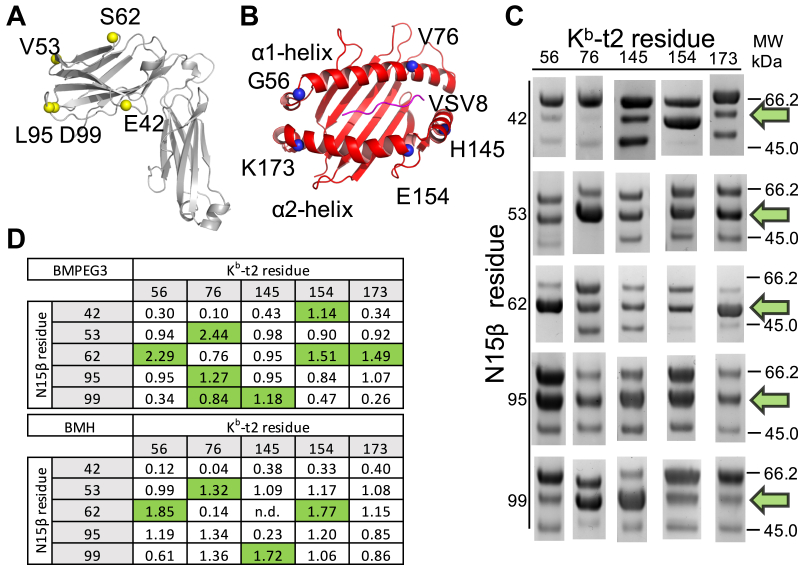
**Survey of the N15β-VSV8/K**^**b**^**-t2 interface using chemical linkage.***A* and *B*, single Cys mutants shown by spheres (*yellow* and *blue*, respectively) on the cartoon presentation of N15β (*A*) in *gray* and VSV8/K^b^-t2 (*B*) in *red* with a *purple* VSV8 peptide were selected for proximity to the putative binding surface for the pairwise linkage screen. *C*, reaction mixtures separated by SDS-PAGE and Coomassie blue stained are shown. The dimeric region of a representative lane for each reaction with BMPEG3 is shown with the heterodimer band denoted with a *green arrow*. Full replicates of reactions with BMPEG3, BMH, and BMB are given in Fig. S2. *D*, the heterodimer specificity (see Supplemental methods) for the BMPEG3 and BMH linkers is denoted in a matrix of reaction combinations. The tables demonstrate that the specific linkage pairs defined by the shorter BMH linker are the subset of the specific linkage pairs found using the longer BMPEG3 linker. Residue pairs with significant specificity, highlighted in *green*, were defined and selected as pairs that exceed the median plus standard deviation within each row, thus correcting for β dimerization, as detailed in Experimental procedures.

We probed the specificity of the crosslinking reactions by generating a series of single Cys mutations on both N15β and VSV8/K^b^-t2. We predicted that the variation in the crosslinking yields could be used in a combinatorial fashion to distinguish among the multitude of possible binding orientations (Figs. 1 and 3, *A* and *B*). In well-mixed solutions with a 1:1 monomer ratio, the statistically predicted homo- and heterodimer distribution after linkage is 1:1:2 (β2:MHC2:β1-MHC1). However, we observed that Cys sites and linker length can each modulate these dimer ratios (Fig. 3). In contrast to fusion protein linkers, chemical linkage not only provides the heterodimer product for further structural studies but also a site and distance-specific measure between the two single Cys mutant proteins. The heterodimer specificity, as defined in the Supplemental methods, equals 1 when the heterodimer ratio follows the statistically predicted random distribution, *i.e.*, no specificity is observed. If the specificity is greater than 1, the heterodimer is formed preferentially; if specificity is lower than 1, the heterodimer formation is disfavored. The latter can be caused by preference for homodimer formation, heteromolecular repulsion between the component protein sites due to steric hindrance, charge distribution, or both. Various linker configurations were tried, and we determined that higher flexibility and length were needed for a more robust survey of linkage sites (Fig. S1). We thus focused on the two linkers utilized in Figure 3, *C* and *D*. Figure 3*D* shows the specificity numbers using the linkers BMPEG3 and 1,6- bis(maleimido)hexane (BMH) for the indicated pairs. To determine the specific linkage pairs, we had to consider that in each case the heterodimer formation competes with the dimerization of N15β. It has been shown previously that N15β forms a dimer with low affinity, and residues 42 to 42’ and 99 to 99’ are located in close proximity within the N15β homodimerization interface (23), which is demonstrated by high β_2_ yields and suppressed heterodimer specificity numbers in rows 1 and 5 on Figure 3*D*. By correcting for the β dimerization as detailed in Experimental procedures, the most consistent “hotspot” pairs highlighted in green are N15β 42:K^b^-t2 154, N15β 53:K^b^-t2 76, N15β 62:K^b^-t2 56/154/173, N15β 95:K^b^-t2 76, and N15β 99:K^b^-t2 76/145. Our result that the specific linkage sites defined by the shorter BMH linker are a subset of the ones found using the longer BMPEG3 linker is consistent with the intuition that linkage reaction yields correlate to interresidue distances. A subset of sites was tested using a third, even shorter bridge length reagent, 1, 4-bis (maleimido) butane (BMB) (Table S1). The specificity numbers were lower for the shorter linkers, which one predicts would be more restrictive in pairing. Overall, we conclude that specific association was readily detected using the bifunctional chemical linkage technique.

### Chemical linkage provides distance restraints *via* bridge length cutoffs and a molecular ruler approach for structural calculations

**Figure 4:**
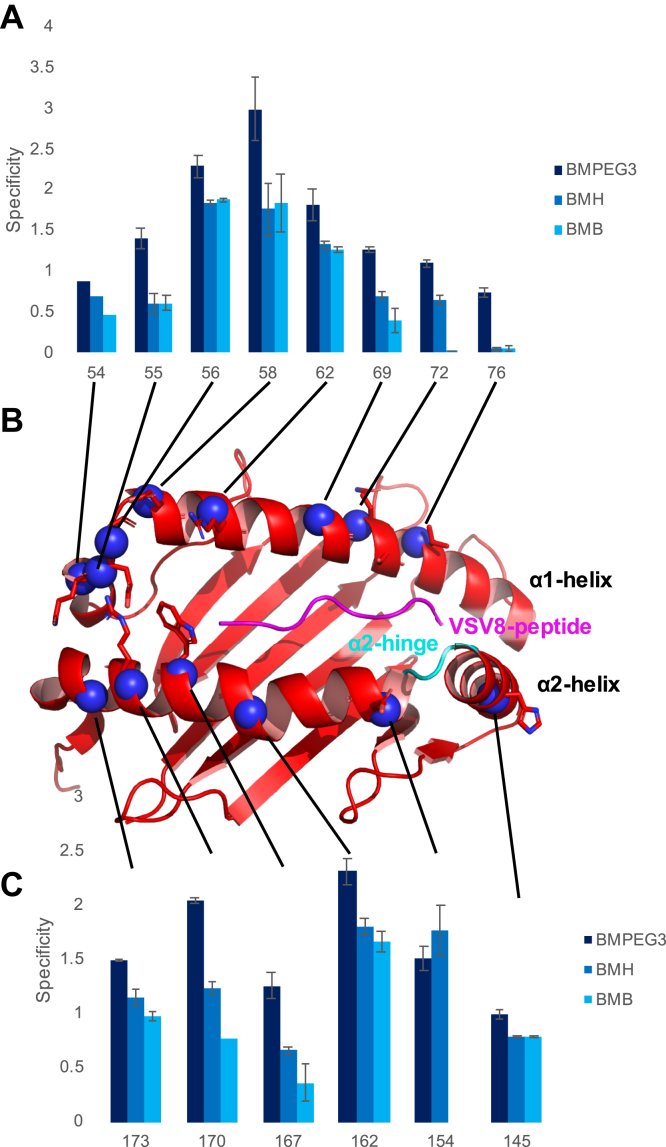
**α-Helices are internal molecular rulers for assessing distances by linkage.***A* and *C*, linkage specificities as detected by SDS-PAGE for residues denoted on the X-axis. Each K^b^-t2 variant was linked to N15β S62C using BMPEG3, BMH, and BMB linkers. SDS-PAGE separations are shown in Fig. S3. *B*, ribbon drawing of K^b^-t2 as observed in crystal structures (PDB ID: 1KPU). The *blue* spheres indicate the single Cys mutations generated in the α1 and α2 helix regions of the K^b^-t2 molecule. The hinge region of the α2 helix (residues 149–153) is shown in *cyan*.

A potential strength of the linkage-based techniques is that residue pairs located close in the heterodimer complex are linked preferentially. For more accurate distance information, in addition to linker compounds with variable bridge lengths, one can use natural distance ladders in the protein structures *per se* (Fig. 4). Structured regions within the MHC, such as the α-helices shown in Figure 4*B*, can be used as an internal molecular ruler. To investigate the consistency of the linkage distance information, we generated consecutive single Cys mutations in the α1 and α2 helix regions of the K^b^-t2 molecule. α-Helices are one of the most ordered secondary structural elements with a distance of 5.4 Å and 3.6 residues per turn (24, 25). Thus, Cys residues in consecutive turns serve as molecular rulers when linked to the same N15β S62C partner. Figure 4*A* demonstrates that residue 58 on the α1-helix shows the highest linkage specificity for BMPEG3 as well as for BMH and BMB. Neighboring residues 56 and 62 also retain specificities greater than 5 standard deviations from the random distribution value of 1 (n = 3) for all three linkers. Although the specificity maximum appears at the same site for all three linkers, specificity decreases progressively faster for the shorter BMH and the shortest BMB.

Linkage data on the α2 helix of K^b^-t2, which contains an MHC-characteristic hinge region (residues 149–153) flanking residue 151, show that specificity measurements for the α2 helix mutants do not follow the same gradual response as seen for α1, apparently reflecting the importance of the side-chain orientation and the break in the helical structure of α2 (Fig. 4*C*). If distance were the only factor in linkage efficiency, one would expect the specificity of 167 to be intermediate between that of 162 and 170, but in fact it is lower than either. It is possible that the direction of the side chain in this case is an overriding factor in the specificity and provides a good contrast to the very regular pattern seen in measurements of the α1 helix (Fig. 4*A*). Similarly, the drop-off in specificity between residue 154 and 145 is more severe than simple distance measurements would predict. The break in the helix is likely responsible (Fig. 4, *B* and *C*). This is evident in the larger difference between 55 and 56 within the α1 helix (Fig. 4, *A* and *B*). To better understand this, it is possible that replacing the direct Euclidean distances with solvent accessible surface distance (SASD), defined in prior studies as the shortest path between two amino acids that does not penetrate the protein surface (26), would better predict the responses shown here and as elaborated in Figs. S6 and S7 later.

### Molecular modeling using chemical linkage distance restraints converges to a single conformation of the preTCR–pMHC in solution

**Figure 5:**
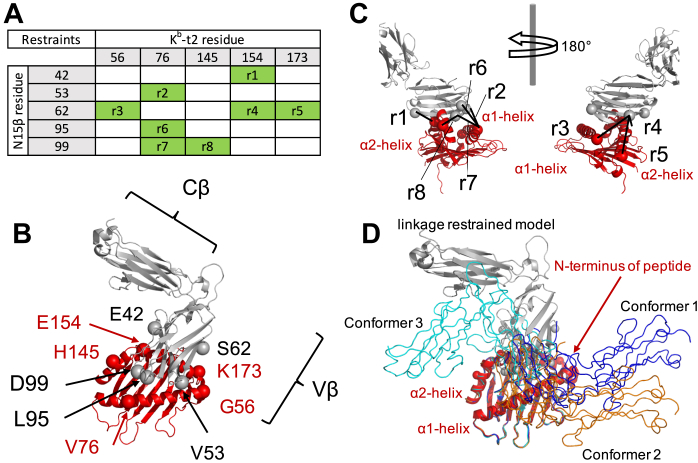
**Molecular modeling of N15β–K**^**b**^**-t2 complex orientation in solution.***A*, the distance restraints r1 to r8 were determined using bifunctional linkage as described in Figure 3. The residue pairs that were identified as proximal using pairwise linkage reactions are highlighted in *green*. *B*, the model generated using restraints r1 to r8 and the previously defined interaction surfaces is shown in cartoon representation in *gray* (N15β) and *red* (K^b^-t2). *C*, restraints are superimposed on the model complex shown in two orientations to demonstrate their compatibility with the model. *D*, superposition of modeled complex onto previously postulated complex candidates (20) with results most similar to but still distinct from Conformer 3. Complexes were aligned according to VSV8/K^b^-t2 position.

As noted above, while residues participating in interaction between N15β and VSV8/K^b^ were identified by NMR cross-saturation or chemical shift perturbation, the docking orientation of the complex could not be unambiguously resolved [(Fig. 1), (18, 20)]. The three lowest energy clusters of binding conformations in which Conformers 1 and 2 utilize a distinct docking orientation from Conformer 3, differing by approximately 180°, could not be further refined due to lack of orientational restraints (Fig. 1*C*, ref (20)). We thus sought to experimentally investigate the lowest energy models of N15β-VSV8/K^b^-t2 predicted by molecular modeling. To this end, we utilized linkage data presented in Figure 3 to orient the N15β-VSV8/K^b^-t2 complex. Linkage specificity (Fig. 3) was interpreted as indicative of spatial proximity between the sites and hence structural restraints were generated using the most specific pairings (Fig. 5*A*). HADDOCK was employed as described in the Experimental procedures with these restraints in addition to those used previously (20). In the identified candidate structures, the Vβ domain had a narrow angle distribution over the pMHC and the solution converged to a single interaction mode (Fig. 5). It appears that the linkage-assisted restrained model is consistent with CDR3 poised over the C-terminus of the VSV8 peptide with Vβ patch and CC’ loop positioned over the α2 helix (Fig. 5*B*). The restraints generated by linkage are highlighted in Figure 5*C* and appear qualitatively consistent with the convergent model. The linkage appears more consistent with Conformer 3 in Figure 1*C*, with variation in the orientation (Fig. 5*D*) while conforming to the transverse approach angle previously suggested (18, 20). To verify the model, we calculated the distances between the Cα atoms of the residues probed by linkage. Fig. S6 shows that for each N15β residue the K^b^-t2 site that showed the highest linkage specificities (Fig. 3) were closest as determined by the shortest SASD using Jwalk (27), which is apparent from the matching shading between the two tables (Fig. 3, Fig. S6), as well. The agreement between the Euclidian distances calculated (Figs. S6 and S7) and specificities was less stringent. While the data from Figure 4 was not used in generating the model, it appears that the SASD calculation for this model is more in line with the linkage restraint-calculated conformation as opposed to either Conformer 1 or 2 from previous calculations (Fig. 1, Fig. S7).

### The local linkage specificity is corroborated by global NMR features

**Figure 6:**
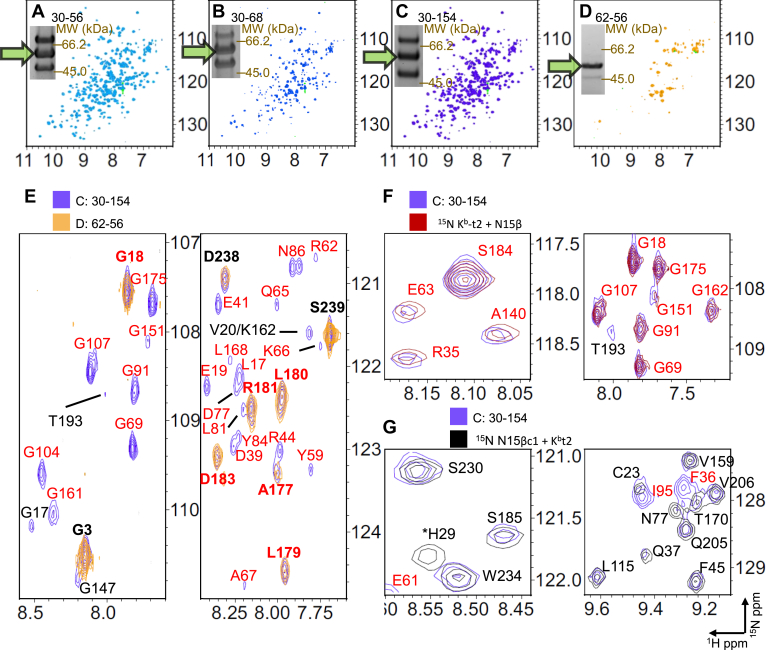
**Solution NMR corroboration of linkage specificity.***A*–*D*, ^1^H-^15^N TROSY-HSQC spectra of the amide region of purified heterodimeric BMPEG3 linked constructs of N15β and VSV8/K^b^-t2: N15β S30C - K^b^-t2 G56C (*A*), N15β S30C - K^b^-t2 K68C (*B*), N15β S30C - K^b^-t2 E154C (*C*), and N15β S62C - K^b^-t2 G56C (*D*) with the final concentrations of 187, 54, 121, and 106 μM, respectively. Each N15β and K^b^-t2 variant was ^15^N labeled, having 328 amino acid residues in total, 309 of which have NMR active amide protons. The number of assigned peaks detected were 274, 254, 267, and 86, respectively. SDS-PAGE insets demonstrate the dimer composition of the BMPEG3 linkage reaction for each linkage site pair. The middle band on the SDS-PAGE (*green arrow*) corresponds to the N15β–K^b^-t2 heterodimer with calculated heterodimer specificities of 0.8, 0.9, 0.8, 1.8. Relevant spectral and sample statistics are tabulated in Table S2. *E*, selected regions of the ^15^N TROSY-HSQC spectra (*C*) and (*D*) were overlaid with peaks remaining in the specific (N15β S62C-K^b^-t2 G56C) spectrum are highlighted in bold. The NMR peaks deriving from N15β and K^b^-t2 residues are labeled in *black* and *red*, respectively. *F*, selected regions of ^1^H-^15^N TROSY-HSQC of spectrum C were superimposed with the spectrum of 200 μM ^15^N K^b^-t2 in the presence of 200 μM unlabeled N15β (*red*). *G*, regions with multiple N15β signals from spectrum C were overlaid with the spectra of 200 μM ^15^N N15β in the presence of 200 μM unlabeled N15β (*black*).

**Figure 7:**
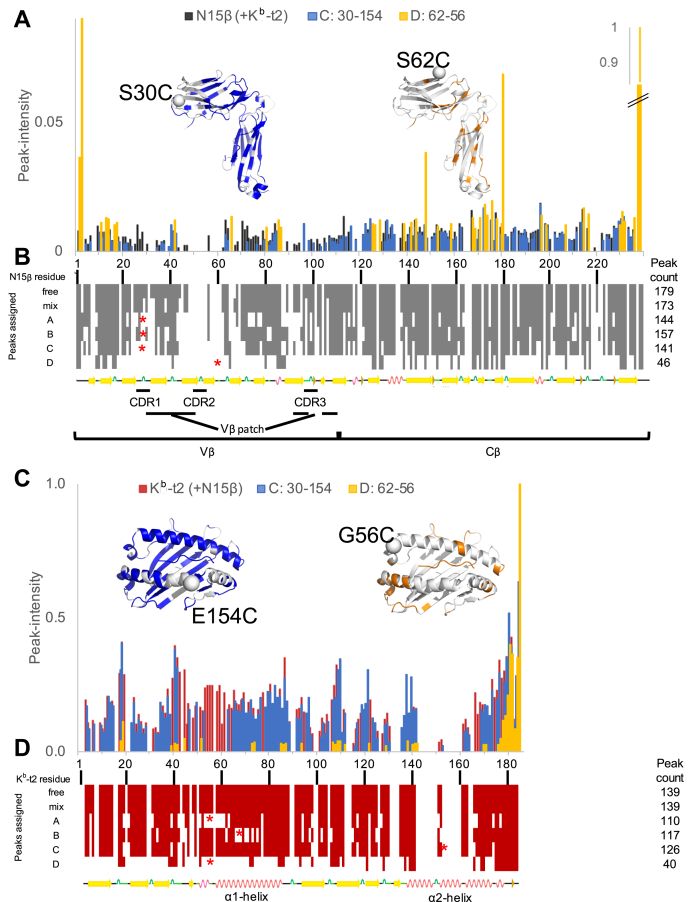
**NMR peak intensity data are structurally correlated to linkage specificity.***A* and *C*, NMR peak intensities for nonspecifically linked N15β S30C - K^b^-t2 E154C (Fig. 6*C*), the specifically linked N15β S62C-K^b^-t2 G56C (Fig. 6*D*), and unlinked N15β-VSV8/K^b^-t2 mixture were plotted *versus* residue number. The NMR intensities were scaled to residues 178 to 184 of K^b^-t2 or residues 230 to 232 of N15β; these reference regions were previously shown to be unaffected by binding (18, 20). Assigned residues that remain detectable in spectra are colored on cartoon inset representations of N15β (*A*) and K^b^-t2 (*C*) for the indicated linked protein components. Labeled spheres show the linkage site. *B* and *D*, the assigned residues are highlighted *gray* and *red*, respectively, on the primary structure of N15β (*B*) and K^b^-t2 (*D*). The linkage sites are denoted by asterisks. The peaks identified for spectra delineated in Figure 6, *A*–*D* are compared with the individual and mixed unlinked N15β and K^b^-t2.

To prove that residue linkage specificity is not only a local feature of the connected protein surfaces, but also related to the global phenomenon of N15β-K^b^-t2 complex formation, we selected four BMPEG3-linked N15β and VSV8/K^b^-t2 pairs for NMR analysis. One sample, N15β S62C-K^b^-t2 G56C, showed high linkage specificity, whereas three others, N15β S30C-K^b^-t2 G56C, -K^b^-t2 K68C, and -K^b^-t2 E154C, were termed nonspecific, as indicated by low heterodimer linkage yields by SDS-PAGE (Fig. 6, *A*–*D* inserts, Table S1). We measured the ^1^H-^15^N TROSY-HSQC spectra of ^1^H-^15^N labeled N15β and ^1^H-^15^N labeled VSV8/K^b^-t2, in which only bound VSV8 was unlabeled. Using the previously published assignments of N15β and K^b^-t (20) we measured spectral changes of both protein components in linked samples and compared them with the nonlinked proteins and their 1:1 mixtures. Spectra of the four linked samples are shown along with the SDS-PAGE separation demonstrating the specificity of the interaction in Figure 6, *A*–*D*. The spectra of the nonspecific site-linked proteins (Fig. 6, *A*–*C*) have more detected peaks compared with proteins linked at the specific site (Fig. 6*D*, Table S2). When proteins are specifically linked, dispersed peaks are no longer detected, with resonances remaining for peaks corresponding to termini or mobile loops (Fig. 7). Resonances detected in nonspecifically linked proteins are nearly identical to those measured in mixtures of unlinked proteins (Fig. 6, *F* and *G*) indicating no changes in overall fold of linked proteins. Peak intensity or peak counts *versus* residue number for N15β (Fig. 7, *A* and *B*) and VSV8/K^b^-t2 (Fig. 7, *C* and *D*) illustrate the effect of linkage site on spectral peak intensity in a structure-specific manner. Figure 7, *A* and *C* compare relative peak intensities of the specifically linked N15β S62C−K^b^-t2 G56C and nonspecific N15β S30C–K^b^-t2 E154C with unlinked proteins. There is preferential loss of intensity in all structured regions for the specifically linked construct, but with more retention of signal in the C-domain of N15β, the domain most distal to the interaction surface within the complex (Figs. 1*A* and 7, *A* and *B*). K^b^-t2 residues affected were widespread with losses throughout the protein, unsurprisingly given the central location of the peptide binding groove in an overall smaller protein (Fig. 7, *C* and *D*). The effect in the nonspecifically linked proteins is more moderate, with retention of intensity in more residues throughout (Fig. 7, *A* and *C*).

It is generally true that peaks present in the nonspecifically linked constructs are a subset of those in mixed unlinked proteins, with additional loss of peaks for Cys-modified residues (Fig. 7, *B* and *D*). The overall spectral changes of nonspecifically linked dimers indicated comparable, but slightly lower affinities between the nonspecifically linked protein components than the unlinked ones. Detailed interaction site information of the specifically linked construct could not be resolved using the TROSY-HSQC spectra due to relaxation-induced signal loss (Figs. 6 and 7). We suggest that the preTCR–pMHC interaction observed by NMR in case of the nonspecifically linked heterodimers is due to a supramolecular heterodimer–heterodimer interaction. In contrast, in the specifically linked heterodimer the preTCR–pMHC interaction preferably appears intramolecularly between the two linked components (Fig. S4), but at a higher occupancy due to the enrichment of appropriately oriented binding partner engendered by the linkage (Fig. 6, *E*–*G*). Nonspecifically linked heterodimers behave as individual proteins, each tethered but tumbling almost independently. However, in the case of the specific heterodimer, the formation of the biological preTCR–pMHC complex is facilitated, which increases the apparent molecular size and rotational correlation time that causes NMR line broadening. Fig. S4 illustrates the hydrodynamic radius (R_H_) difference between the monomeric N15β, VSV8/K^b^-t2 and their complexes as calculated applying the Burchard’s approximation (28) on the atomic coordinates from crystallography or molecular modeling (see the Fig. S4 legend). Consistent with the notion that the smaller molecular weight and more spherical K^b^-t2 experiences a larger hydrodynamic radius increase during complexation (33% *versus* 21% for N15β, Fig. S4), in the K^b^-t2 the relative peak intensity losses for the detected peaks were more evident than the intensity loss for the residues present in the N15β spectra when the proteins are specifically linked (Fig. 7, Fig. S4). Note also that the line broadening at the interface may increase due to changes in the overall exchange rate, most likely in the association rates due to the increased local availability of ligand.

### Crystallization of linked β-pMHC

Due to the weak interaction of N15β chain with VSV8/K^b^-t2 at thermal equilibrium, initial attempts to cocrystallize the nonlinked proteins were unsuccessful. Although protein crystals formed readily in the mixtures, the presence of both components in the same crystal was not observed. To promote the cocrystal formation, a crystallographic screening trial was initiated using the BMPEG3-linked construct of N15β S62C and K^b^-t2 G56C. Proteins were expressed, purified, refolded, and linked, then purified for crystallography by two successive steps of SEC, as the yield of heterodimer was high enough that IEC was unnecessary. Needle-shaped crystals grew within 1 to 2 weeks to final dimensions of about 0.70 × 0.02 × 0.01 mm (Fig. S5), and preliminary data suggest diffraction to 3.3 Å. Crystal structure analysis of the linked protein constructs will be detailed in a separate paper.

### Interaction geometry of unlinked β-pMHC is verified by PCS NMR as an orthogonal approach

**Figure 8:**
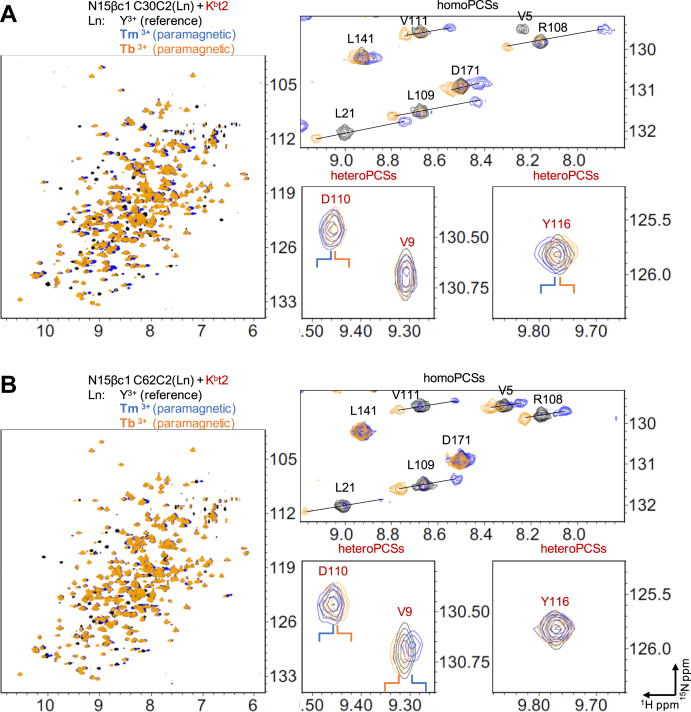
**Homo- and heteroPCSs as observed in the NMR spectra of N15β and VSV8/K**^**b**^**-t2 mixtures.**^1^H-^15^N TROSY-HSQC spectral overlay of a mixture of ^15^N labeled H2K^b^-t2 bound to unlabeled VSV8 and ^15^N labeled N15β. The C2-tag coordinated with Y^3+^ (*black*), Tb^3+^ (*blue*), or Tm^3+^ (*orange*) was linked to residues S30C (*A*) and S62C (*B*) of N15β. Expanded views of the full spectra highlight homomolecular PCSs observed on residues of N15β (*black*) and heteroPCSs on K^b^-t2 residues (*red*). Selected diamagnetic cross-peaks are labeled with their resonance assignments and connected by lines with their paramagnetic partners. N15β and VSV8/K^b^-t2 were mixed in 1:1 ratio, the final concentration was 70 uM for the N15β C30C2 samples, and 140 μM for the N15β C62C2 samples.

The NMR results described above are consistent with a site-specific enhancement of a pre-existing binding interface engendered with the bis-maleimide linkage strategy. However, to bolster the evidence for the orientation described in Figure 5, an orthogonal measurement was used. Unlinked proteins with paramagnetic centers generate appropriate pseudocontact shifts (PCSs) that can be applied for structural determination if the paramagnetic component is site-specifically attached to the target protein in a rigid manner (29). In the present study, describing the interaction between N15β and K^b^-t2 relies on PCS values determined for the C2-tagged (30) protein itself (homoPCS) and for the protein interaction partner (heteroPCS) in the same solution. The C2 tag loaded with lanthanide (Ln) ions [see Ref. (30) and Experimental procedures] was attached to single Cys mutants of N15β or K^b^-t2 *via* disulfide linkage at one of several sites. Robust data were generated using mutants S30C and S62C of N15β but could not be generated for N15β G16C or S181C or VSV8/K^b^-t2 R79C or H145C, possibly due to excessive mobility of the C2 moiety on those sites. Figure 8 demonstrates the observed PCSs by overlaying the spectra of N15β-K^b^-t2 mixtures, which only differed in the Ln ions (Ln: Y^3+^, Tb^3+^ and Tm^3+^) coordinated by the C2-tag. Homo- and heteroPCSs were identified in the same experiment since the ^1^H-^15^N TROSY-HSQC spectra were recorded using ^15^N labeled protein components N15β C30C2(Ln) or C62C2(Ln), ^15^N labeled K^b^-t2, and unlabeled VSV8. Sections of the same spectra focus on a region with representative homoPCSs for Tb^3+^ and Tm^3+^ and heteroPCSs of V9, D110, and Y116 of VSV8/K^b^-t2. The chemical shifts of the C2(Y^3+^) tagged N15β and K^b^-t2 were similar to those of the nontagged proteins published previously (18, 20) because the diamagnetic tag does not generate a PCS effect and hence served as a control for the paramagnetic Tb^3+^ and Tm^3+^ adducts. As illustrated in Figure 8, the PCSs for the same residues measured with different Ln ions were situated along straight lines in superimposed spectra, Tb^3+^ and Tm^3+^ being on either side of Y^3+^. Since the amide proton and nitrogen are spatially close, the PCS effect is correlated in the ^1^H and ^15^N dimensions (X- and Y-axes, respectively) [Fig. 8, (31)]. The PCS effect was thus assessed using only the ^1^H chemical shift changes.

**Figure 9:**
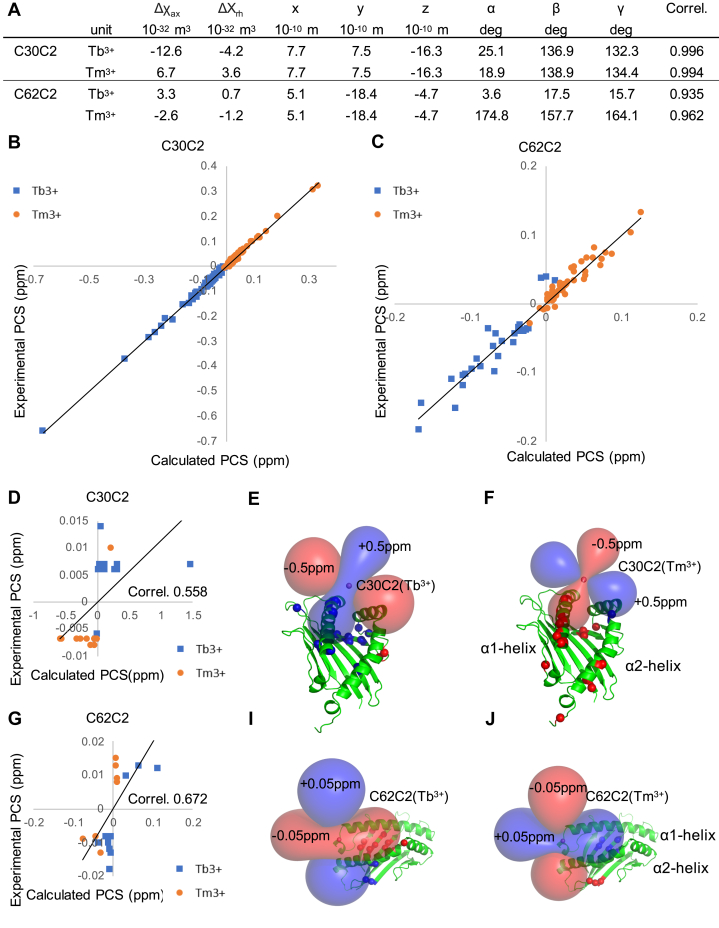
**Verification of the linkage model using PCSs observed in the N15β–VSV8/K**^**b**^**-t2 mixture.** The C2(Ln)-tag (Ln: Y^3+^, Tb^3+^, or Tm^3+^) was attached to residues of 30 (*A*, *B*, *D*, *E*, *F*) or 62 (*A*, *C*, *G*, *I*, *J*) of N15β. *A*, Tb^3+^ and Tm^3+^ induced ^1^H homoPCSs of the backbone amide protons (HN) of N15β were used to fit the homoΔχ-tensors simultaneously to a common position (x, y, z) by the program Paramagpy (47) using the linkage model structure of N15β–K^b^-t2 complex. The distance between the O atom of the Ser residues 30 and 62 of N15β and the corresponding tensor positions is 7.6 and 1.0 Å, respectively. The last column reports the correlation between observable and back-calculated homoPCSs. The heteroPCSs of the K^b^-t2 were not used to fit the homoΔχ-tensors. *B* and *C*, correlation plots between observable homoPCSs (vertical axis, in ppm) and back-calculated PCSs (horizontal axis, in ppm) were produced by the appropriate tensors on *A*. *D* and *G*, the experimental heteroPCSs were plotted against the back-calculated PCSs using the homoΔχ-tensors on *A* and the atomic coordinates of the linkage model of the N15β–VSV8/K^b^-t2 complex. Correlation between each experimental and calculated heteroPCSs is indicated. *E*, *F*, *I* and *J*, ribbon diagrams of K^b^-t2 demonstrate its orientation in the linkage model relative to the homoΔχ-tensors. Residues that exhibit heteroPCSs (<−0.06 ppm, *red spheres* or >0.06 ppm, *blue spheres*) are indicated. The homoΔχ-tensors are represented as PCS isosurfaces (±0.5 ppm for site 30, ±0.05 ppm for site 62).

The homoΔχ-tensor parameters of site 30 and 62 of N15β are listed in Figure 9*A* along with the correlation values between the experimental and back-calculated homoPCSs (Tables S3–S6). The calculated paramagnetic center coordinates were in each case within 8 Å to the tagged residue, and tensor fitting converged to a single solution. The identified homoPCSs derived mostly from the Cβ domain, since both tags were located on the Vβ. The NMR peaks of the Vβ domain were lost partly because of the paramagnetic relaxation enhancement [PRE, (29)] in the proximity of the tagging site, and partly because of the chemical exchange due to the K^b^-t2 complex formation. The inherent conformational dynamics of the β chain subdomains Vβ and Cβ (32, 33) lead to ensemble-averaged paramagnetic effects and contributed to the uncertainty of the homoΔχ-tensor; however, the overall correlation (Fig. 9, *B* and *C*) offered a rational basis for evaluating the complex docking.

The orientation of the N15β chain with respect to the K^b^-t2 was coded into the anisotropy of the observed heteroPCSs. To validate our linkage model of the N15β/K^b^-t2 complex, we back-calculated the PCSs for the amide protons of K^b^-t2 using the homoΔχ-tensors determined based on only the PCSs of N15β in Figure 9*A*. Figure 9, *D* and *G* show the correlation plots between the experimental and back-calculated PCSs for the amide protons of K^b^-t2 in the linkage model of N15β-K^b^-t2 (Table S7). As shown in Figure 9, *D* and *G*, the observed heteroPCSs were about ten times smaller than the back-calculated and homoPCSs (0.5–0.1 *versus* 0.05–0.01 ppm for ^1^H, Fig. 9), which is in line with the weak affinity in solution, *K*_D_ = 400 μM, determined previously by NMR titration (18). Therefore, about 25% of each protein is complexed in a 200-200 μM mixture.

HomoΔχ-tensors make an oriented fingerprint of N15β on the structure of K^b^-t2 that allows interpretation of heteroPCSs. Figure 9, E, F, I, and *J* show red and blue lobes representing the homoPCS isosurfaces describing the orientation of N15β with respect to K^b^-t2. According to our linkage model, the C2(Tm^3+^) tag on site 30 of N15β is located close to the middle of the α1 helix of K^b^-t2 (Fig. 9, *E* and *F*), which is consistent with the data that most significant heteroPCSs were observed on the α1 helix. In agreement with our model, the orientation of homoΔχ-tensors on site 30 resulted in a mostly positive heteroPCS (blue) in case of Tb^3+^ and a mostly negative heteroPCSs (red) for Tm^3+^ (Fig. 9, I and *J*). For tagging site 62 the observed heteroPCSs were localized on the N terminal part of the peptide binding groove of K^b^-t2 (beginning of the α1 helix and the end of α2 helix) (Fig. 9, I and *J*), and the sign of the heteroPCSs also correlated with the orientation of the homoΔχ-tensors in the linkage model. The experimental heteroPCSs in N15β-K^b^-t2 mixtures showed moderate positive correlation with the linkage model and with Conformer 3 of Figure 1*C*, while the heteroPCS data sets were not correlated or moderately negatively correlated with Conformers 1 and 2 (Fig. 9, *D* and *G*, Fig. S8 and Table S7).

### Linkage specificity analysis demonstrates peptide selectivity in the preTCR–pMHC interaction

**Figure 10:**
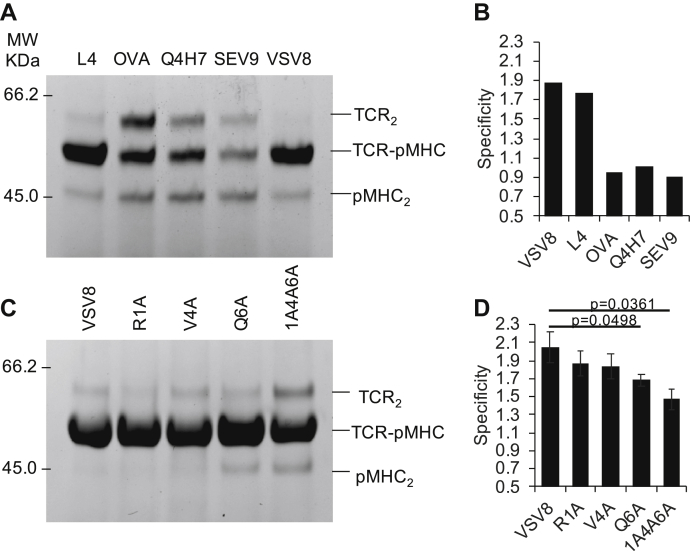
**Specificity of peptide discrimination by N15β.***A*, linkage screen by SDS-PAGE of N15β S62C and K^b^-t2 G56C bound to peptides VSV8, L4, OVA, Q4H7, or SEV9 using BMPEG3 linker. *B*, quantification of the peptide dependence of the N15β S62C–K^b^-t2 G56C heterodimer specificity on SDS-PAGE in (*A*) as detailed in Experimental procedures; *C*, a representative figure of the BMPEG3 linkage screen of N15β S62C and K^b^-t2 G56C bound to VSV8 peptide variants; *D*, quantification of the heterodimer specificity of N15β S62C and K^b^-t2 G56C bound to VSV8 peptide variants using BMPEG3 linker in four parallel linkage experiments with the bars representing standard deviation. Additional data for *C* and *D* is in Fig. S3. *p* values are as determined by one-tailed Student’s *t*-test.

The N15αβ TCR exhibits exquisite specificity for the peptide RGYVYQGL (VSV8) bound to K^b^, with single amino acid differences, such as the mutation of residue Val4 to Leu (L4), leading to both dramatically decreased activation of mature N15αβ TCR bearing T cells and differential developmental outcome of N15αβ thymocytes (34, 35, 36). The peptide specificity of the N15 preTCR, while appearing to be less stringent than the N15αβTCR, has not been systematically probed (18). By examining crosslinking specificity ratios using a BMPEG3 linkage screen of N15β S62C and K^b^-t2 G56C bound to peptide variants, it appears that the N15β preTCR recognizes VSV8 and its L4 variant similarly but not three unrelated peptides: the K^b^-restricted SIINFEKL epitope of ovalbumin (OVA), the OVA variant SIIQFEHL (Q4H7), or the Sendai virus peptide FAPGNYPAL (SEV9), presented by K^b^-t2 in solution (Fig. 10, *A* and *B*). SDS-PAGE analysis of the heterodimer distributions demonstrates that the heterodimer ratio returns to the statistically predicted nonspecific ratios in the case of those three irrelevant peptides (Fig. 10*B*). We also performed alanine scanning of VSV8 to evaluate the critical peptide residues in the preTCR–pMHC interface (Fig. 10, *C* and *D*). When each of the upward-facing p1, p4, and p6 residues of peptides bound in the groove of K^b^-t2 (R1, V4, Q6) were mutated to Ala individually as single mutations (R1A, V4A, Q6A), or together as a triple mutant (1A4A6A), loss in specificity occurred mainly as a result of the C-terminal Q6A change. Although a single mutation of R1 or V4 had no significant impact on reactivity individually, the triple mutant showed the most difference from WT (Fig. 10, *C* and *D*). Even so, 1A4A6A still manifest some specificity, in contrast to the unrelated peptides OVA, Q4H7, and SEV9 (Fig. 10, Fig. S9). While the shorter linkers do not discriminate readily between single mutants and WT, 1A4A6A reproducibly reacts less than WT with each linker (Fig. S9). These results demonstrate that perturbations in interacting protein affinities can be monitored effectively by linkage specificity without the necessity of *a priori* high-resolution structural information.

## Discussion

Single-chain fusions have facilitated structure determination of several weakly bound complexes (37, 38), including crystal structures of TCR–pMHC complexes (39), single-chain VαVβ TCR (40), CD3εγ, and CD3εδ (41, 42). In each case, the use of “crosslinking” by creation of a single polypeptide linked by flexible residue intermediaries was used to stabilize an existing weak interaction sufficiently for study by crystallographic or NMR methods, leading to significant insights into the biological function of the molecules in question. Herein, we have used a bifunctional crosslinker to achieve similar stabilization, but with the added advantage of freely choosing the site of crosslinking through introduction of a Cys residue in each of the two interaction partners. We demonstrate the stabilization effect by a construct with high crosslinking specificity, defined as higher preTCR–pMHC yields than that in a statistically predicted distribution. Additionally, we demonstrate that chemical linkage provides site and distance-specific information between the two single Cys mutant proteins and is useful in providing distance restraints in molecular docking calculations. As in the aforementioned cases, verification of the structural data through orthogonal methods is critical, as is cross-validation of models within the technique. In this regard, we report a solution docking model based on linkage distance restraints that is also supported by using independent PCS NMR data generated *via* paramagnetically labeled, nonlinked protein partners. Our results are consistent with previous NMR data (18, 20) in that multiple sites comporting with our model show significant crosslinking specificity, while those that would disprove our model do not (Figure 3, Figure 4, Figure 5). The solution docking presented here also points to the C-terminal part of the K^b^-bound VSV8 peptide as playing a key role in specific peptide recognition, which was further clarified by alanine screening (Fig. 10). Significantly, the specificity of the crosslinking reaction depends on the presence of a compatible peptide (Fig. 10, *A* and *B*), which is entirely consistent with our model of the preTCR–pMHC interaction and assures that we are not simply kinetically trapping nonspecific complexes.

The use of the bifunctional bis-maleimide linkers presented here provides several advantages over existing methods. First, the reaction and its interpretation may be carried out from start to finish using standard wet lab equipment for protein purification and SDS-PAGE. Second, there are several choices for linker composition, which may be varied for desired length, stiffness, or other biophysical characters. Third, information gleaned through modulation of linkage sites as well as linker composition may be used in structural analysis for more detailed insights into the interaction in question. If component structures are well characterized, more definitive structural models may be produced by combining linkage information with molecular modeling. This strategy thus enables an intermediate throughput, low-cost structural investigative tool.

The experiments in Figure 10 exemplify the potential power of this technique. Here, we were able to measure the effect of peptide variation on preTCR–pMHC recognition. From the data shown here, the recognition of the peptide, while having an impact on preTCR binding, may be considerably less stringent than that of the αβTCR, indicating a binding interface for preTCR–pMHC that differs significantly from that of the αβTCR–pMHC. The elimination of specificity with the three unrelated peptides OVA, Q4H7, and SEV9 (Fig. 10) in the absence of bioforces may be through introduction of steric clashes rather than, or in addition, to loss of defined contacts. Using optical tweezers, it has been shown previously that under force the N15preTCR, like the αβTCR, discriminates among different peptides bound to the same K^b^ molecule *via* a catch bond mechanism. The bond lifetime of the N15preTCR–VSV8/K^b^ interaction lengthened with increasing force with a peak lifetime at 10 to 20 pN. The N15preTCR also formed a strong catch bond with Q4H7/K^b^, while its catch bond with OVA/K^b^ was weak and was not observable for SEV9/K^b^ (21). Our preTCR–pMHC linkage screen provides further details on the differential ligand sensitivity of a preTCR–pMHC interaction, which is tuned by bioforces under biological conditions. Similarly, it may be advantageous to use these techniques to probe novel β chains and mutational variants thereof to demonstrate the generality of the preTCR–pMHC paradigm that is now arising. If a single interaction mode as defined in Figure 5 is dominant for the preTCR, then a survey of β chains could be completed with a single reaction for each preTCR–pMHC pairing. Alternatively, it is possible that the binding mode is general but with variability in twist, tilt, and shift previously observed with the αβTCR (17).

Lastly, this technology provides a convenient method for optimizing constructs and providing substrates for X-ray crystallographic or NMR structural studies. Despite over a decade of efforts to isolate a preTCR–pMHC complex, the present strategy is the first to successfully do so. Encouragingly, we have been able to isolate not only the N15β–VSV8/K^b^ (Figs. 2 and 4), which we have studied extensively, but also several peptide variants bound to K^b^ (Fig. 10). This study has also led to the formation of diffraction quality crystals of the N15β–VSV8/K^b^ complex (Fig. S5). The ability to choose incorporation sites relieves requirement for coincident N and C termini for the constituent domains, expanding the possible targets for such an approach. One could proceed *de novo* with a global screen of sites or start with functional or preliminary structural data, as we have herein, to narrow the linkage search space. It seems likely that the crosslinking methodology described here would also be useful in combination with single-molecule electron microscopy in elucidating transient macromolecular binding complexes. Since many protein–protein interactions are inherently weak and transient, the broad potential for a general method of capturing transient states through this application for structural study is substantial.

### Note added in proof

Since this paper was submitted, an X-ray crystallographic structure of the N15preTCRβ–pMHC complex has been solved by our collective team (43) using the methodology described herein for large-scale protein production, linkage, and purification. Those structural data are in full agreement with the current conclusions from linkage distance restraints, NMR, and PCS data. Collectively, our results show that the β chain in the preTCR binds to the C-terminal segment of an MHC-bound peptide, as does the β chain in the mature TCRαβ heterodimer, while employing a distinct docking mode.

## Experimental procedures

### Protein expression and purification

N15β, K^b^-t2, and their variants were produced as detailed (20) with modifications for protecting the free Cys residue. All protein constructs were cloned into pET11d expression vector (New England BioLabs Inc). Single Cys variants were constructed by site-directed mutagenesis using standard protocols (Invitrogen). Recombinant plasmids were cloned using *E. coli* strain One Shot MAX Efficiency DH5α-T1 R (Thermo Fisher Scientific). The protein expression host *E. coli* strain was One Shot BL21 Star (DE3) (Thermo Fisher Scientific). Unlabeled or isotopically labeled proteins were expressed into inclusion bodies (ib) using LB (unlabeled, 25 g/l Luria broth, Sigma, 100 mg/l Carbenicillin) or M9 (^15^N labeled; 50 mM Na_2_HPO_4_, 20 mM KH_2_PO_4_, 10 mM NaCl, 18 mM ^15^NH_4_Cl, 2 g/l Glucose, 2 mM MgSO_4_, 20 μM CaCl_2_, 100 mg/l Carbenicillin, 10 μM FeCl_2_, 2 ml Vitamin Cocktail).

N15β used in this study denotes the N15β-c1 (F128R/V144Q/L146Q) mutant designed for decreased self-association *via* the C-module as described in Ref. (20). N15β chain ib preparations were washed thoroughly in 50 mM Tris-Cl, pH 8.0, 150 mM NaCl (TBS), and TBS +1% Triton X-100 (TBS-T), dissolved in 6M Guanidine-HCl, and refolded by dilution in 5.4 M Guanidine-HCl, 0.1 M Tris-HCl pH 8.0, 1M arginine, 1 mM reduced glutathione (GSH), and 0.1 mM oxidized glutathione (GSSG) and subsequent dialysis in TBS for >16 h.

K^b^-t2 is a single residue, C121Q, mutant of the truncated K^b^-t described in (20). The octapeptides VSV8, vesicular stomatitis virus octapeptide (RGYVYQGL); L4, VSV8 variant (RGYLYQGL); OVA ovalbumin derivative (SIINFEKL); Q4H7, OVA variant (SIIQFEHL), and SEV9, Sendai virus peptide (FAPGNYPAL) were chemically synthesized (United Biosystems, Inc, Herndon, VA, USA), the identity confirmed by MS, and the purity of >95% was verified by HPLC. The ib preparations of the K^b^-t2 variants were washed in TBS and TBS-T and dissolved in 8M urea. K^b^-t2 and peptide were mixed in 2:1 mass ratio and diluted in 20 mM Tris-Cl, pH 8.0, 8M urea buffer containing 1 mM reduced glutathione (GSH) and 0.1 mM oxidized glutathione (GSSG) and dialyzed serially against 2M, 1M, 0.5 M, and 0M urea in 20 mM Tris-Cl, pH 8.0 for 2 h or overnight for each step with a final additional dialysis against 0M urea, 20 mM Tris-Cl, pH 8.0 overnight. All proteins were purified by successive rounds of SEC.

### Covalent linkage of heterodimers

Each protein (30 μM) containing a single nondisulfide bonded Cys was prepared for linkage by reduction using 25 mM (for β chains) or 5 mM (for K^b^-t2) dithiothreitol (DTT) in phosphate buffered saline (PBS, pH 7.4) for 30 min at 25 °C. After reduction the protein pairs were mixed at a 1:1 ratio and immediately separated *via* analytical SEC (Superdex S200A, GE Healthcare Life Sciences) to remove the DTT. The corresponding fractions were collected immediately, concentrated to 15 μM (of each subunit), and the appropriate bismaleimide linker (Fig. S1): (1,11-bis(maleimido)triethylene glycol (BMPEG3); 1,8-bis(maleimido)diethylene glycol (BMPEG2); 1,6-bis(maleimido)hexane (BMH); 1,4-bis(maleimido)butane (BMB); 1,2-bis(maleimido)ethane (BME); N,N'-1,4-phenylenebismaleimide (oPBM); N,N'-1,3-phenylenebismaleimide (mPBM); N,N'-1,2-phenylenebismaleimide (pPBM)) was added at 30 to 45 μM (two-three times excess), or as appropriate for each experiment at 25 °C. The mixture was incubated for 20 min and analyzed by reducing SDS-PAGE as detailed in Supplemental methods.

For NMR studies the BMPEG3-linked heterodimers were purified in two chromatographic steps (Fig. 2). The dimeric components were purified from the monomeric forms by SEC (Superdex S200A, GE Healthcare Life Sciences; PBS, pH 7.4). The dimer mixture from SEC was separated by ion exchange chromatography (IEC; MonoQ HR 5/5, GE Healthcare Life sciences). IEC peaks were eluted using multistep programmed ionic strength gradient starting from 100% buffer A (20 mM Tris-HCl, pH 8.0) to 50% buffer B (20 mM Tris-HCl + 1.0 M NaCl, pH 8.0) though a gradient volume of 30 ml. The purity of the “ab” heterodimer peak was verified by SDS-PAGE (Fig. 2*C*), the heterodimer fractions were concentrated and exchanged to NMR buffer (10% D_2_O/H_2_O, 50 mM Na-phosphate, 150 mM NaCl, pH 7.0) using a centrifugal filter unit (Amicon Ultra with a MWCO of 10 kDa; Millipore).

### Protein tagging for PCS NMR

Single-cysteine mutants of N15β (G16C, S30C, S62C, S181C) and K^b^-t2 (R79C, H145C) were prepared as uniformly ^15^N-labeled samples. The C2-tag [2,20,200-(10-(2-Oxo-2-(2-(pyridin-2-yldisulfanyl)ethylamino)-ethyl)-1,4,7,10-tetraazacyclododecane-1,4,7-triyl)tris(N-((R)-1-phenylethyl)acetamide)] loaded with the Tb^3+^, Tm^3+^, or Y^3+^ (30, 44) was attached to one or another of the single-cysteine mutants by adding the protein to a threefold excess of the respective metal complexed C2 and incubating at room temperature for 18 h. The excess of C2 was eliminated, and the proteins were exchanged into NMR buffer (10% D_2_O/H_2_O 50 mM Na-phosphate, 150 mM NaCl, pH 7.0) using a centrifugal filter unit (Amicon Ultra with a MWCO of 10 kDa; Millipore, Billerica, USA). Final protein concentrations were between 70 and 200 μM in 1:1 mixture of each as determined by UV absorbance at 280 nm prior to mixing (70 uM for the N15β C30C2 samples, and 140 μM for the N15β C62C2 samples).

### NMR spectroscopy

NMR spectra were recorded of the uniformly ^15^N-labeled solutions of the protein mixtures or linked preparations of N15β and K^b^-t2 variants in NMR buffer at 25 °C, using standard ^1^H-^15^N- TROSY-HSQC pulse sequences on Bruker 750 MHz spectrometer equipped with a TCI cryoprobe, Varian 600 MHz spectrometer equipped with cryogenically cooled HCN triple resonance probe, or a Bruker 500 MHz spectrometer with a room temperature probe. All spectra were acquired with Topspin (Bruker) or VNMRJ (Varian) and processed with NMRPipe (45) and visualized using CARA (46).

### Pseudocontact shift (PCS) determination

The PCS effects were monitored in the ^1^H-^15^N-TROSY-HSQC spectra of the N15β and K^b^-t2 1:1 protein mixture. Utilizing the backbone assignments of N15β and K^b^-t2 (18, 20), PCSs for the backbone amide protons (^1^H) observed in ^1^H-^15^N-TROSY-HSQC spectra were determined in ppm as chemical shifts measured in the presence of a paramagnetic lanthanide (Tb^3+^ and Tm^3+^) minus the chemical shift observed in the presence of diamagnetic Y^3+^. The error of the PCS values was estimated by the sum of the errors in peak position due to random noise as determined by nmrPipe (45). Only homoPCSs were used for homoΔχ-tensor fitting. HeteroPCSs were used for analysis if their absolute value reached 0.06 ppm.

### Crystallization

All protein samples were concentrated to 8 mg/ml in the 0.5× PBS buffer. The commercial crystallization kits including Index Screen (Hampton Research, Aliso Viejo, CA, USA), JCSG Core Suites (QIAGEN, Hilden, Germany), and Top96 (Anatrace, Maumee, OH, USA) were used for initial crystal sorting. The screening for crystallization conditions was set up with a Formulatrix NT8 robot using the sitting drop vapor diffusion technique in INTELLI-PLATE 96 Well (Art Robbins Instruments) in the Longwood Center for Structural and Chemical Biology at Dana-Farber Cancer Institute. For each condition, 0.1 μl of protein and 0.1 μl of crystallization formulation were mixed; then the mixture was equilibrated against 50 μl of the crystallization solution in each reservoir well.

### Structure building for molecular modeling and visualization

The models for N15β and K^b^-t2 used crystal structures for N15β and VSV8/K^b^ (PDB ID: 3Q5Y, 1KPU) and were used for the molecular docking, tensor fitting, hydrodynamic radii calculations, and for creating the figures. Chain A served as a model for the monomeric N15β. The models of K^b^-t2 used residues 1 to 185 of chain A and chain P for the VSV8 peptide. Amide hydrogens were added with PyMOL (version 2.0 Schrödinger, LLC.) to build the search models for the PCSs-based magnetic susceptibility tensor fitting.

### Magnetic susceptibility tensor fitting

PCSs for the backbone amide protons (^1^H) observed in ^1^H-^15^N-TROSY-HSQC spectra were determined in ppm as chemical shifts measured in the presence of a paramagnetic lanthanide (Tb^3+^ and Tm^3+^) minus the chemical shift observed in the presence of diamagnetic Y^3+^. Eight parameters (x_i_, y_i_, z_i_, ΔΧ_ax_, ΔΧ_rh_, α, β, and γ) corresponding to the atomic coordinates of the complex structure were determined from each set of PCS values using the program Paramagpy (47). PCSs follow the equation:

$${\text{PCS}}_{\text{i}}=1/\left(12\text{π}r_{i}^{3}\right)\left[\Delta X_{\text{ax}}\left({2\text{z}}_{\text{i}}^{2}-{\text{x}}_{\text{i}}^{2}-{\text{y}}_{\text{i}}^{2}\right)/\left(r_{i}^{2}\right)+\;1.5\Delta X_{\text{rh}}\left({\text{x}}_{\text{i}}^{2}-{\text{y}}_{\text{i}}^{2}\right)/\left(r_{i}^{2}\right)\right]$$

where x_i_, y_i_, z_i_ are the Cartesian coordinates of the amide protons of residue i in the ΔΧ-tensor frame, *r*_i_ is the distance of the nuclear spin i from the paramagnetic center, ΔΧ_ax_ and ΔΧ_rh_ are the axial and rhombic components of the ΔΧ-tensor. The orientation of the ΔΧ-tensor frame with respect to the protein frame was specified by three Euler angles α, β, and γ. Tb^3+^ and Tm^3+^ PCSs of the same tagging site were used simultaneously to fit a common position, but varied magnitude and orientation of Δχ-tensor.

### Molecular modeling

Molecular docking using chemical linkage data for N15β and K^b^-t2 interaction was carried out using HADDOCK2.2 Web Server (48). The input data consisted of the N15β and VSV8/K^b^-t2 models described above; ambiguous restraints for the interaction interface and the unambiguous distance restraints. In total, 2% of the ambiguous restraints were randomly excluded (less than or equal to 1 restraint), while 1000 complex geometries were generated for rigid body docking in five consecutive iteration steps. 180° rotated solutions were also sampled during rigid body energy minimalization. In total, 200 lowest energy structures were used for semiflexible refinement. Consecutively, structures were solvated in a shell of TIP3P water; the water-mediated contacts between amino-acid pairs defined from the Kyte-Doolittle hydrophobicity scale; and rigid-body docking were performed for solvated complexes.

The interaction interface residues (N15β: 94, 31, 102, 27, 41, 49, 6, 44, 104, 32, 36, 14, 101, 113, 97, 96, 98; K^b^-t2: 26, 66, 70, 73, 74, 116, 123, 124, 151, 152, 162, 163) were defined as published in (20) determined by combined chemical shift changes of the ^1^H-^15^ N TROSY-HSQC NMR spectra. Distance restraints were determined by pairwise linkage of single Cys variants of N15β (E42C, V53C, S62C, L95C, D99C) and K^b^-t2 (G56C, V76C, H145C, E154C, K173C) using BM(PEG)_3_ linker (See Fig. 3). Three parallel linkage mixtures were analyzed by SDS PAGE, and the specificity of heterodimer formation for each was calculated. Median and standard deviation of the linkage specificities were determined for each N15β variant (median of each row of Fig. 3*D*). K^b^-t2–N15β residue pairs were defined as having specificities one standard deviation higher than the median specificity for each linker type independently; however, only BMPEG3 data was used for restraint generation. Cα–Cα distance restraints were defined using Crystallography and NMR system (CNS) syntax by lower and upper margin of 3 to 23 Å. Within this range, the potential energy of the restraint was zero as implemented in HADDOCK (48, 49).

## Data availability

All data are contained within the article and the Supporting information.

## Conflict of interest

The authors declare that they have no conflicts of interest with the contents of this article.
