# Supplementary material for: A general chemical crosslinking strategy for structural analyses of weakly interacting proteins applied to preTCR–pMHC complexes
Source: J Biol Chem. 2021 Jan 8;296:100255. doi: 10.1016/j.jbc.2021.100255 (PMC7948749; doi:10.1016/j.jbc.2021.100255)
Supplement: Supplemental Figures S1–S9 and Tables S1, S2 and S7 [file mmc1.docx]

| Table S3 | | |
| --- | --- | --- |
| N15β | PCS / ppm | |
| residue | 30C2-Tb^3+^ | |
|  | Calc. | Exp. |
| 10 | -0.223 | -0.206 |
| 12 | -0.125 | -0.119 |
| 15 | -0.068 | -0.07 |
| 16 | -0.067 | -0.056 |
| 17 | -0.067 | -0.077 |
| 21 | -0.26 | -0.262 |
| 22 | -0.369 | -0.37 |
| 28 | -0.007 | -0.007 |
| 35 | -0.666 | -0.661 |
| 65 | -0.121 | -0.115 |
| 78 | -0.133 | -0.147 |
| 83 | -0.078 | -0.083 |
| 84 | -0.089 | -0.104 |
| 85 | -0.113 | -0.107 |
| 87 | -0.196 | -0.211 |
| 108 | -0.282 | -0.282 |
| 109 | -0.235 | -0.24 |
| 110 | -0.158 | -0.152 |
| 111 | -0.123 | -0.123 |
| 113 | -0.066 | -0.054 |
| 115 | -0.054 | -0.047 |
| 117 | -0.055 | -0.049 |
| 118 | -0.059 | -0.061 |
| 119 | -0.065 | -0.051 |
| 122 | -0.053 | -0.048 |
| 123 | -0.048 | -0.041 |
| 124 | -0.046 | -0.042 |
| 125 | -0.036 | -0.031 |
| 126 | -0.036 | -0.033 |
| 127 | -0.028 | -0.024 |
| 129 | -0.027 | -0.034 |
| 132 | -0.022 | -0.01 |
| 133 | -0.022 | -0.014 |
| 134 | -0.019 | -0.01 |
| 135 | -0.019 | -0.008 |
| 141 | -0.036 | -0.034 |
| 142 | -0.037 | -0.044 |
| 144 | -0.048 | -0.04 |
| 145 | -0.061 | -0.056 |
| 146 | -0.054 | -0.061 |
| 147 | -0.056 | -0.058 |
| 148 | -0.063 | -0.065 |
| 149 | -0.072 | -0.076 |
| 152 | -0.11 | -0.106 |
| 153 | -0.121 | -0.13 |
| 155 | -0.106 | -0.103 |
| 156 | -0.076 | -0.067 |
| 157 | -0.068 | -0.072 |
| 158 | -0.047 | -0.055 |
| 159 | -0.037 | -0.034 |
| 160 | -0.024 | -0.034 |
| 161 | -0.026 | -0.027 |
| 163 | -0.042 | -0.04 |
| 168 | -0.058 | -0.071 |
| 169 | -0.06 | -0.062 |
| 171 | -0.079 | -0.082 |
| 173 | -0.1 | -0.107 |
| 174 | -0.107 | -0.123 |
| 176 | -0.072 | -0.083 |
| 177 | -0.062 | -0.062 |
| 178 | -0.056 | -0.068 |
| 180 | -0.054 | -0.055 |
| 181 | -0.062 | -0.061 |
| 182 | -0.068 | -0.071 |
| 183 | -0.073 | -0.074 |
| 185 | -0.072 | -0.07 |
| 186 | -0.059 | -0.061 |
| 188 | -0.045 | -0.047 |
| 189 | -0.044 | -0.049 |
| 191 | -0.025 | -0.037 |
| 193 | -0.017 | -0.016 |
| 194 | -0.018 | -0.008 |
| 195 | -0.02 | -0.014 |
| 196 | -0.016 | -0.011 |
| 197 | -0.015 | -0.006 |
| 199 | -0.011 | -0.001 |
| 200 | -0.013 | -0.014 |
| 202 | -0.024 | -0.016 |
| 203 | -0.035 | -0.034 |
| 204 | -0.04 | -0.04 |
| 205 | -0.057 | -0.051 |
| 206 | -0.061 | -0.06 |
| 207 | -0.088 | -0.09 |
| 209 | -0.108 | -0.108 |
| 210 | -0.088 | -0.097 |
| 212 | -0.076 | -0.067 |
| 215 | -0.054 | -0.048 |
| 217 | -0.033 | -0.028 |
| 223 | -0.035 | -0.033 |
| 225 | -0.053 | -0.048 |
| 226 | -0.072 | -0.076 |
| 228 | -0.051 | -0.06 |
| 230 | -0.042 | -0.043 |
| 231 | -0.043 | -0.033 |
| 232 | -0.03 | -0.032 |
| 233 | -0.027 | -0.028 |
| 234 | -0.02 | -0.026 |
| 235 | -0.017 | -0.028 |
| 236 | -0.017 | -0.021 |
| 237 | -0.013 | -0.007 |
| 238 | -0.011 | -0.007 |
| 239 | -0.013 | -0.006 |

| Table S4 | | |
| --- | --- | --- |
| N15β | PCS / ppm | |
| residue | 30C2-Tm^3+^ | |
|  | Calc. | Exp. |
| 10 | 0.115 | 0.114 |
| 11 | 0.093 | 0.103 |
| 12 | 0.064 | 0.066 |
| 13 | 0.056 | 0.055 |
| 14 | 0.038 | 0.039 |
| 15 | 0.032 | 0.034 |
| 16 | 0.03 | 0.026 |
| 17 | 0.031 | 0.029 |
| 18 | 0.035 | 0.028 |
| 21 | 0.125 | 0.114 |
| 22 | 0.188 | 0.204 |
| 28 | 0.011 | 0.011 |
| 35 | 0.318 | 0.31 |
| 64 | 0.019 | 0.028 |
| 65 | 0.04 | 0.04 |
| 66 | -0.001 | -0.001 |
| 72 | -0.004 | -0.005 |
| 77 | 0.065 | 0.06 |
| 80 | 0.026 | 0.017 |
| 83 | 0.035 | 0.033 |
| 84 | 0.04 | 0.04 |
| 85 | 0.051 | 0.056 |
| 86 | 0.061 | 0.069 |
| 87 | 0.096 | 0.1 |
| 106 | 0.335 | 0.322 |
| 108 | 0.145 | 0.14 |
| 109 | 0.117 | 0.122 |
| 110 | 0.079 | 0.082 |
| 111 | 0.06 | 0.067 |
| 112 | 0.043 | 0.049 |
| 113 | 0.032 | 0.027 |
| 115 | 0.027 | 0.027 |
| 117 | 0.028 | 0.022 |
| 118 | 0.03 | 0.027 |
| 119 | 0.034 | 0.036 |
| 123 | 0.025 | 0.027 |
| 124 | 0.024 | 0.032 |
| 125 | 0.019 | 0.018 |
| 126 | 0.019 | 0.022 |
| 127 | 0.015 | 0.014 |
| 129 | 0.015 | 0.014 |
| 132 | 0.012 | 0.011 |
| 133 | 0.012 | 0.013 |
| 135 | 0.011 | 0.007 |
| 138 | 0.012 | 0 |
| 139 | 0.015 | 0.014 |
| 141 | 0.02 | 0.027 |
| 142 | 0.02 | 0.009 |
| 144 | 0.025 | 0.023 |
| 145 | 0.032 | 0.03 |
| 146 | 0.029 | 0.025 |
| 147 | 0.029 | 0.027 |
| 148 | 0.033 | 0.029 |
| 149 | 0.037 | 0.027 |
| 151 | 0.056 | 0.061 |
| 152 | 0.057 | 0.059 |
| 153 | 0.063 | 0.066 |
| 155 | 0.057 | 0.056 |
| 156 | 0.04 | 0.041 |
| 157 | 0.037 | 0.038 |
| 158 | 0.025 | 0.022 |
| 159 | 0.02 | 0.013 |
| 160 | 0.013 | 0.011 |
| 161 | 0.013 | 0.014 |
| 163 | 0.022 | 0.022 |
| 164 | 0.028 | 0.023 |
| 168 | 0.032 | 0.034 |
| 169 | 0.033 | 0.034 |
| 171 | 0.043 | 0.043 |
| 173 | 0.054 | 0.059 |
| 174 | 0.057 | 0.059 |
| 176 | 0.037 | 0.041 |
| 177 | 0.032 | 0.039 |
| 178 | 0.029 | 0.034 |
| 180 | 0.027 | 0.029 |
| 181 | 0.032 | 0.038 |
| 182 | 0.035 | 0.041 |
| 183 | 0.038 | 0.038 |
| 185 | 0.038 | 0.045 |
| 186 | 0.032 | 0.029 |
| 188 | 0.025 | 0.027 |
| 189 | 0.024 | 0.027 |
| 191 | 0.014 | 0.017 |
| 192 | 0.011 | 0.014 |
| 193 | 0.01 | 0.006 |
| 194 | 0.01 | 0.011 |
| 195 | 0.011 | 0.007 |
| 196 | 0.009 | 0.006 |
| 197 | 0.008 | 0.007 |
| 199 | 0.005 | -0.001 |
| 200 | 0.007 | 0.007 |
| 202 | 0.013 | 0.02 |
| 203 | 0.018 | 0.022 |
| 204 | 0.021 | 0.028 |
| 205 | 0.03 | 0.028 |
| 206 | 0.032 | 0.038 |
| 207 | 0.046 | 0.055 |
| 208 | 0.043 | 0.032 |
| 209 | 0.056 | 0.064 |
| 210 | 0.046 | 0.054 |
| 212 | 0.04 | 0.047 |
| 214 | 0.03 | 0.035 |
| 215 | 0.029 | 0.027 |
| 217 | 0.017 | 0.015 |
| 223 | 0.018 | 0.02 |
| 225 | 0.028 | 0.033 |
| 226 | 0.038 | 0.047 |
| 227 | 0.037 | 0.043 |
| 228 | 0.027 | 0.026 |
| 229 | 0.03 | 0.036 |
| 230 | 0.022 | 0.016 |
| 231 | 0.023 | 0.015 |
| 232 | 0.016 | 0.021 |
| 233 | 0.014 | 0.02 |
| 234 | 0.01 | 0.014 |
| 236 | 0.009 | 0.007 |
| 237 | 0.007 | 0.007 |
| 238 | 0.006 | 0.006 |
| 239 | 0.007 | 0.006 |

| Table S5 | | |
| --- | --- | --- |
| N15β | PCS / ppm | |
| residue | 62C2-Tb^3+^ | |
|  | Calc. | Exp. |
| 2 | -0.026 | -0.037 |
| 3 | -0.032 | -0.04 |
| 7 | -0.076 | -0.035 |
| 11 | -0.091 | -0.08 |
| 12 | -0.065 | -0.044 |
| 19 | -0.022 | -0.035 |
| 22 | -0.068 | -0.098 |
| 24 | -0.069 | -0.061 |
| 26 | -0.058 | -0.055 |
| 27 | -0.029 | -0.039 |
| 29 | -0.04 | -0.043 |
| 37 | -0.168 | -0.184 |
| 93 | -0.097 | -0.095 |
| 94 | -0.086 | -0.092 |
| 97 | -0.063 | -0.076 |
| 104 | -0.11 | -0.118 |
| 106 | -0.106 | -0.103 |
| 108 | -0.11 | -0.104 |
| 109 | -0.165 | -0.144 |
| 110 | -0.125 | -0.109 |
| 111 | -0.119 | -0.151 |
| 151 | -0.042 | -0.056 |
| 152 | -0.036 | -0.034 |
| 153 | -0.034 | -0.031 |
| 174 | -0.024 | -0.036 |
| 214 | 0.012 | 0.034 |
| 215 | 0.001 | 0.04 |
| 217 | -0.006 | 0.037 |

| Table S6 | | |
| --- | --- | --- |
| N15β | PCS / ppm | |
| residue | 62C2-Tm^3+^ | |
|  | Calc. | Exp. |
| 2 | 0.023 | 0.023 |
| 3 | 0.027 | 0.029 |
| 7 | 0.047 | 0.062 |
| 12 | 0.019 | 0.031 |
| 13 | 0.037 | 0.047 |
| 15 | 0.038 | 0.055 |
| 17 | 0.051 | 0.036 |
| 21 | 0.002 | -0.007 |
| 24 | 0.055 | 0.067 |
| 26 | 0.053 | 0.047 |
| 27 | 0.026 | 0.027 |
| 28 | 0.026 | 0.013 |
| 29 | 0.037 | 0.034 |
| 42 | 0.03 | 0.033 |
| 72 | 0.004 | 0.009 |
| 73 | 0.01 | 0.024 |
| 74 | 0.08 | 0.075 |
| 85 | 0.073 | 0.056 |
| 87 | 0.126 | 0.134 |
| 95 | 0.053 | 0.041 |
| 97 | 0.062 | 0.068 |
| 106 | 0.087 | 0.072 |
| 108 | 0.074 | 0.066 |
| 109 | 0.113 | 0.104 |
| 110 | 0.064 | 0.082 |
| 112 | 0.035 | 0.053 |
| 113 | 0.022 | 0.02 |
| 114 | 0.019 | 0.027 |
| 115 | 0.013 | -0.005 |
| 117 | 0.011 | 0.006 |
| 119 | 0.01 | 0.005 |
| 123 | 0.005 | 0.006 |
| 125 | 0.004 | 0.005 |
| 132 | 0.003 | 0.005 |
| 135 | 0.003 | 0.006 |
| 138 | 0.003 | -0.006 |
| 140 | 0.004 | 0.006 |
| 142 | 0.005 | 0.005 |
| 145 | 0.008 | 0.007 |
| 146 | 0.007 | 0.006 |
| 147 | 0.008 | 0.007 |
| 148 | 0.009 | 0.008 |
| 149 | 0.011 | 0.013 |
| 151 | 0.024 | 0.028 |
| 152 | 0.02 | 0.022 |
| 153 | 0.021 | 0.022 |
| 155 | 0.015 | 0.012 |
| 156 | 0.01 | 0.01 |
| 158 | 0.007 | 0.009 |
| 159 | 0.006 | 0.007 |
| 163 | 0.007 | 0.007 |
| 168 | 0.008 | 0.007 |
| 169 | 0.008 | 0.007 |
| 171 | 0.011 | 0.009 |
| 173 | 0.014 | 0.014 |
| 174 | 0.017 | 0.02 |
| 176 | 0.009 | 0.014 |
| 177 | 0.008 | 0.006 |
| 178 | 0.007 | 0.008 |
| 180 | 0.007 | 0.013 |
| 181 | 0.009 | 0.019 |
| 182 | 0.01 | 0.011 |
| 183 | 0.01 | 0.013 |
| 185 | 0.01 | 0.013 |
| 186 | 0.008 | 0.006 |
| 190 | 0.005 | 0.005 |
| 191 | 0.004 | 0.006 |
| 192 | 0.003 | 0.007 |
| 197 | 0.003 | 0.005 |
| 202 | 0.004 | 0.005 |
| 203 | 0.005 | 0.007 |
| 205 | 0.007 | 0.007 |
| 206 | 0.007 | 0.007 |
| 207 | 0.011 | 0.014 |
| 209 | 0.015 | 0.015 |
| 210 | 0.008 | 0.014 |
| 212 | -0.008 | -0.007 |
| 215 | -0.021 | -0.029 |
| 223 | -0.002 | -0.008 |
| 227 | 0.005 | 0.005 |
| 228 | 0.003 | 0.006 |
| 231 | 0.005 | 0.007 |
| 234 | 0.003 | 0.009 |
| 235 | 0.003 | 0.013 |
