# Supplementary material for: A general chemical crosslinking strategy for structural analyses of weakly interacting proteins applied to preTCR–pMHC complexes
Source: J Biol Chem. 2021 Jan 8;296:100255. doi: 10.1016/j.jbc.2021.100255 (PMC7948749; doi:10.1016/j.jbc.2021.100255)
Supplement: Supplemental Tables S3–S6 [file mmc2.pdf]

Supporting information for

A general chemical crosslinking strategy for structural analyses of weakly interacting proteins applied to preTCR-pMHC complexes

Réka Mizsei, Xiaolong Li, Wan-Na Chen, Monika Szabo, Jia-huai Wang, Gerhard Wagner, Ellis L. Reinherz, Robert J. Mallis

The Supporting information includes:

Supplemental Methods

Figures S1 to S9

Tables S1 to S7

Supplemental figures

Figure S1. Bis-maleimide crosslinkers tested for efficacy in crosslinking studies. Crosslinker names and maximum bridge lengths are indicated above the chemical structures. SDS-PAGE separation of reaction mixtures of N15β S62C and VSV8/K^b-t2 G56C utilizing each linker shown to the left and above each gel region. 0.5, 1 or 2 molar equivalents of each linker versus each protein at 1:1 ratio of proteins. Positions of molecular weight markers 66.2 kDa and 45 kDa are shown (Low Range SDS-PAGE Molecular Weight Standards, Bio-Rad, Hercules, CA, USA). Green arrow denotes the position of the linked heterodimer product. Linkage screens were conducted using 3x excess of linker unless otherwise indicated.

Figure S2. SDS-PAGE separation of linkage reactions using BMPEG3, BMH, and BMB. (A) Reaction replicate lanes of data shown in Fig. 3C**. (B-C)** Reaction replicate lanes of SDS-PAGE of BMH (**B**) or BMB (**C**) linkage of constructs shown in Fig. 3A-B. Each block shows independent reactions that were repeated 1-4 times, no exclusion criteria were used. Data are summarized in Fig. 3D. Positions of molecular weight markers 66.2 kDa and 45 kDa are shown (Low Range SDS-PAGE Molecular Weight Standards, Bio-Rad, Hercules, CA, USA). Green arrow indicates the linked heterodimer product.

Figure S3. SDS-PAGE separation of linkage reactions using BMPEG3, BMH, and BMB for testing the helical ruler. (A-C) Reaction replicate lanes of data shown in Fig. 4A, C**.** Reaction replicate lanes of SDS-PAGE of BMPEG (A) BMH (B) and BMB (C) linkage are shown. Each block shows independent reactions, no exclusion criteria were used. Molecular weights were referenced to Low Range SDS-PAGE (66.2 kDa, 45 kDa) or Precision Plus Protein™ Dual Color (50 kDa, 75kDa) Molecular Weight Standards (Bio-Rad, Hercules, CA, USA). Positions of molecular weight markers 66.2 kDa and 45 kDa are shown. Green arrow indicates the linked heterodimer product.

Figure S4. Hydrodynamic radii (R_H) of N15β and VSV8/K^b-t2 and their linked constructs. The red and gray ellipses represent the monomeric protein components with the interaction surfaces highlighted in yellow. The hydrodynamic radii (R_H) were estimated using the spherical approximation $\frac{R_G}{R_H} = \sqrt{\frac{3}{5}}$. by Burchard (30), where R_G is the gyration radius determined from the pdb coordinates by home-made python script using equation $R_G^2 = \sum_{i,j,k} \sum_{a=1}^{N} \frac{m_a}{M} \left(r_{ai}^2 - R_i^2 \right)$, where r_a are the atomic coordinates, m_a are atomic masses M is the molar mass of the molecule: $M = \sum_{a=1}^{N} m_a$. R denotes the coordinates of the center of mass: $R = \frac{1}{M} \sum_{a=1}^{N} m_a r_a$. Atomic coordinates and masses were extracted from pdb files (crystal structures of monomeric N15β and K^b-t2 were processed as described in Experimental procedures, the linkage model of N15β-K^b-t2 as described in Figure 5). R_H values are visualized as blue circles and are listed in the bottom (in nm) together with the molecular weights (MW) in kDa. If the linkage facilitates the complex formation (specific linkage) the linked constructs behave as single molecules with larger R_H, while monomers linked nonspecifically will have similar hydrodynamic properties to the free monomer components.

Figure S5. Crystals of VSV8/K^b-t2 G56C - BM(PEG)3 - S62C N15β. Crystals grew after initial screening by the sitting-drop vapor-diffusion technique in the presence of 0.100 M Bince pH 9.0, 15% (w/v) PEG 20,000 at 293K. The bar represents 200 µm. Images were obtained using the Rock Imager (Formulatrix Inc., Waltham, Massachusetts, USA) with visible (**A**) and UV (**B**) light, the latter for detection of protein content.

Figure S6. Solvent Accessible Surface Distances (SASD) and Euclidean distances between VSV8/K^b-t2 and N15β residues probed by linkage. (**A, B**) tables show distances between Cα atoms of reaction combinations calculated for our refined model using Jwalk (29). The bottom 10% of the values (closest distances) are highlighted in red for each row (for each N15β residue). (**C**) Residue pair combinations used

as distance restraints (r1-r8) for the model building are highlighted in green (Fig. 3). The colorful tubes on the surface (**D**) and ribbon (**E**) representation of the N15β-VSV8/K^b-t2 complex visualize the shortest SAS path between the residue pairs listed in the table as determined by Jwalk. Structures and residue labels for N15β and VSV8/K^b-t2 are colored gray and red respectively. (**E**) emphasizes only SAS paths between residue pairs selected for model building (r1-r8 as in **C**).

Figure S7. Solvent Accessible Surface Distances (SASD) vs. Euclidean distances between N15β S62C and selected VSV8/K^b-t2 residues. (**A**) Distances between Cα atoms were calculated for our linkage model using Jwalk (29). The colorful tubes on the surface representation of the N15β-VSV8/K^b-t2 complex visualize the shortest SAS path between N15β S62C (labeled black) and VSV8/K^b-t2 residues listed in table (**A**) as determined by Jwalk. **(B)** N15β and VSV8/K^b-t2 are shown in gray and red, respectively. The two views present the α1 and α2 sides of the complex. On each side the most distant K^b-t2 residues are labeled in red. The experimental linkage yields of the corresponding residue pairs are shown in Fig. 4 and Fig. S3.

Figure S8. Correlation between experimental and calculated heteroPCSs for different N15β-VSV8/K^b-t2 docking geometries. (**A**) Correlation plots as per Fig 9D and G, but determined using Conformer1-3. of Figure 1C. (**B**) The correlation of the four docking models with the experimental heteroPCSs are listed. The homoΔχ-tensors in Figure 9A were used for the computations. The strength of the linear association between experimental and back-calculated data sets in **Table S3-6** was quantified by the correlation coefficient determined in MS Excel. Despite the approximately tenfold difference between the experimental and back-calculated PCSs, scaling was not used as correlation coefficient is insensitive for changes in scale or units of measurement.

Figure S9. Specificity of peptide discrimination by N15β. (A) Linkage screen of N15β S62C and K^b-t2 G56C bound to the Ala mutants of VSV8 separated by SDS-PAGE full replicates of Figure10C-D are shown. Positions of 66.2 kDa and 45.0 kDa molecular weight standards are indicated (Low Range SDS-PAGE Molecular Weight Standards, Bio-Rad, Hercules, CA, USA). The linkage reactions were carried out using bifunctional linkers BMB, BMH and BMPEG3. **(B)** Quantification of the heterodimer specificity of N15β S62C and K^b-t2 G56C on SDS-PAGE in (A), using BMB, row 1; scatter plots show individual data points for the two experiments. **(C)** Quantification of the heterodimer specificity of N15β S62C and K^b-t2 G56C on SDS-PAGE in (A), BMH, row 2. Error bars show standard deviation.

Supplemental tables

Table S1. Linkage specificity for the are N15β-VSV8/K^b-t2 complex formation as probed by the BMB linkers and detected on SDS-PAGE. Residue pairs with significant specificity are highlighted in green as per Figure 3.

Table S2. Parameters for ^{1}H-^{15}N TROSY-HSQC spectra of purified heterodimeric linked constructs of N15β and VSV8/K^b-t2. Sample and spectral parameters are listed for ^{1}H-^{15}N TROSY-HSQC spectra of ^{1}H-^{15}N labeled N15β and VSV8/K^b-t2 (Fig. 6 and Fig. 7), in which only bound VSV8 was unlabeled. In the first four rows data for BMPEG3 linked constructs N15β S30C - K^b-t2 G56C (30-56), N15β S30C - K^b-t2 K68C (30-68), N15β S30C - K^b-t2 E154C (30-154) and N15β S62C - K^b-t2 G56C (62-56) are listed. In the last four rows are values for four unlinked NMR samples: free N15β (TCR), N15β in 1:1 mixture with K^b-t2 (TCR+), K^b-t2 (MHC) and K^b-t2 in 1:1 mixture with N15β (MHC+). Reference spectra were recorded as previously published in (21). The sample concentrations are given as measured by absorbance at 280 nm. The noise levels of the HSQC spectra were determined in nmrPipe (47). The average intensities of the reference regions are listed (residues 178-184 for K^b-t2 or residues 230-232 for N15β). Signal to noise (S/N) was calculated dividing the reference peak intensities by the noise level in the spectra. The total number of amide resonances assigned in the spectra are listed, the maximum observable peaks are indicated in

parentheses. In case of the linked N15β-VSV8/K^b-t2 constructs the last column shows the linkage specificity determined by SDS-PAGE. NAN indicates the undefined parameters.

Table S3. HomoPCSs of backbone amide protons of in N15β C30C2(Tb^{3+})-VSV8/K^b-t2 mixtures. N15β S30C mutant was tagged with either C2(Tb^{3+}) (paramagnetic sample) or C2(Y^{3+}) (diamagnetic reference). PCSs were measured as the ^{1}H chemical shifts observed with C2(Tb^{3+}) minus the ^{1}H chemical shifts observed with C2(Y^{3+}). Back-calculated PCSs were obtained from fitting the corresponding homoΔχ-tensor in Figure 9A to the linkage structure.

Table S4. HomoPCSs of backbone amide protons of in N15β C30C2(Tm^{3+})-VSV8/K^b-t2 mixtures. Data are tabulated as in Table 3, but listed for C2(Tm^{3+}) tagged at residue 30 of N15β.

Table S5. HomoPCSs of backbone amide protons of in N15β C62C2 (Tb^{3+})-VSV8/K^b-t2 mixtures. Data are tabulated as in Table 3, but listed for C2(Tb^{3+}) tagged at residue 62 of N15β.

Table S6. HomoPCSs of backbone amide protons of in N15β C62C2(Tm^{3+})-VSV8/K^b-t2 mixtures. Data are tabulated as in Table 3, but listed for C2(Tm^{3+}) tagged at residue 62 of N15β.

Table S7. HeteroPCSs of backbone amide protons in N15β-VSV8/K^b-t2 mixtures. The experimental and back-calculated heteroPCSs are given for amide protons of K^b-t2 residues listed in the first column. The four sections of the table differed in the tagging site (30 or 62) or the lanthanide ion (Ln: Tb^{3+} or Tm^{3+}) complexed by the C2 tag. Each dataset was referenced to the corresponding C2(Y^{3+}) tagged sample. The heteroPCSs were back-calculated using the homoΔχ-tensors in Figure 9A, and the atomic coordinates of the linkage model and Conformer1-3 in Figure 1C.

Supplemental methods

SDS-PAGE

For SDS-PAGE analysis protein mixtures were diluted by 30% in Reducing Sample Buffer (250 mM DTT, NuPAGE LDS Sample Buffer, Invitrogen, Carlsbad, CA, USA), then heated for 2 minutes at 95 °C, cooled and loaded 10 µl/well on 4-12% Bis-Tris gradient gels (Invitrogen, Carlsbad, CA, USA, NuPAGE, Cat. No.: NP0323BOX). The molecular weights were referenced to Low Range SDS-PAGE or Precision Plus Protein™ Dual Color Molecular Weight Standards (Bio-Rad, Hercules, CA, USA)), visualized by Coomassie blue R-250 dye (Imperial™ Protein Stain, Fisher Scientific, Hampton, NH, USA), imaged using Image Lab™ Touch 1.1.0.4, (Bio-Rad).

Obtaining the molar distribution of reaction mixtures

The protein concentration ratios in the linkage reaction mixtures were calculated from the band intensities observed on the SDS-PAGE using Image Lab™ 6.0.0 Software (Bio-Rad). For each lane the position of five bands were selected manually; then the band boundaries and the background subtraction were adjusted automatically. The band intensities were extracted as percentage of the band's volume compared to all band volumes in the lane (Band%). To derive a measure that is proportional to the protein concentrations the SDS-PAGE band intensity values were divided by the calculated molecular weight of the given construct as band intensities are proportional to the molecular mass rather than the molarity. In the calculations below a, b and aa, ab, bb denote the molecular weight corrected band intensities for the monomeric and linked dimeric constructs (Fig. 2A).

For quality assurance two additional SDS-PAGE experiments were carried out. Monomer mixing ratios were monitored before each linkage, which was kept around 50-50%. The maleimide reactivity of the Cys residues were assayed by addition of 1000 fold excess of Methoxypolyethylene glycol maleimide, with an average molecular weight of 5 KDa, resulting in a 5KDa upshift of the monomer masses. The Cys reactivities were always over 70%; and the ratios of reactive Cys bearing monomers were also approximately 1:1.

Definition of specificity by measurement of linkage yields.

To quantify our linkage screen data, we defined a specificity measure for ALB heterodimer formation between proteins A and B. The value of specificity depends on experimental factors such as the affinity between A and B, the location of the reactive amino acid site introduced and the nature of the bifunctional crosslinker (L).

The following irreversible bimolecular reactions were considered to be induced by the addition of the bifunctional crosslinker:

$$A + L \xrightarrow{k1} AL$$

$$\mathrm{B} + \mathrm{L} \xrightarrow{k2} \mathrm{BL}$$

$$\mathrm{A} + AL \xrightarrow{k3} ALA$$

$$\mathrm{A} + \mathrm{BL} \xrightarrow{k4} ALB$$

$$\mathrm{B} + \mathrm{AL} \xrightarrow{k5} ALB$$

$$\mathrm{B} + \mathrm{BL} \xrightarrow{k6} BLB$$

The specificity of the ALB heterodimer formation was defined as a ratio between the observed ALB concentration and expected ratios of ALB heterodimers (ALB_0) in case of non-specific linkage:

$$Linkage\ Specificity = \frac{[ab]}{[ALB_0]}$$

ALB_0 was calculated using the observed monomer concentrations (a, b), and the homodimer concentrations (aa, bb) as shown in Figure 2A.

$$ALB_0 = \frac{[aa]}{[b]} + \frac{[bb]}{[a]}$$

The heterodimer non-specificity was defined as affinity of A to A is the same as to B ($k3$=$k4$) and affinity of B to A is the same as to B ($k5$=$k6$). We also monitored for the homodimer non-specificity (affinity of A to A is the same as B to B; $k3$=$k6$), and linker non-specificity (affinity of linker to A is the same as to B; $k1$=$k2$). In case of N15β the inherent homodimerization could not be neglected for certain residues on the homodimer interface, however, the k_6 kinetic constant for homodimerization should be the same if the same residue of N15β is reacted with the linker. Therefore, when we tested the linkage of the same single Cys N15β residue with several K^b-t2 residues, the increase in “ab” heterodimer yields were indicative for the preferential reactivity of N15β to the given K^b-t2 variant.

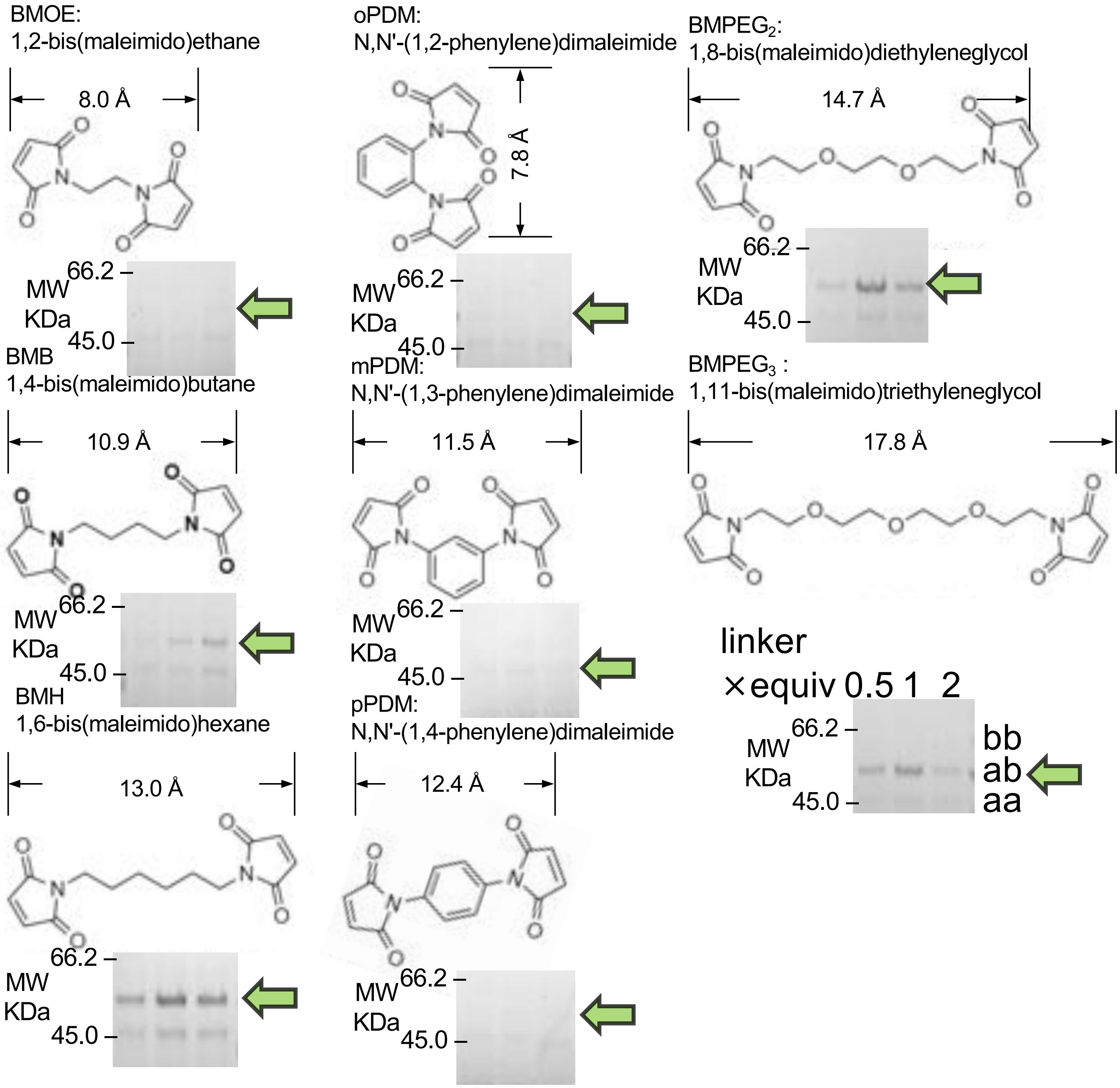
BMOE:
1,2-bis(maleimido)ethane
8.0 Å
oPDM:
N,N'-(1,2-phenylene)dimaleimide
7.8 Å
BMPEG$_2$:
1,8-bis(maleimido)diethyleneglycol
14.7 Å
MW KDa 66.2 45.0
BMB
1,4-bis(maleimido)butane
10.9 Å
mPDM:
N,N'-(1,3-phenylene)dimaleimide
11.5 Å
BMPEG$_3$:
1,11-bis(maleimido)triethyleneglycol
17.8 Å
BMH
1,6-bis(maleimido)hexane
13.0 Å
pPDM:
N,N'-(1,4-phenylene)dimaleimide
12.4 Å
linker
×equiv 0.5 1 2
bb
ab
aa

Figure S1

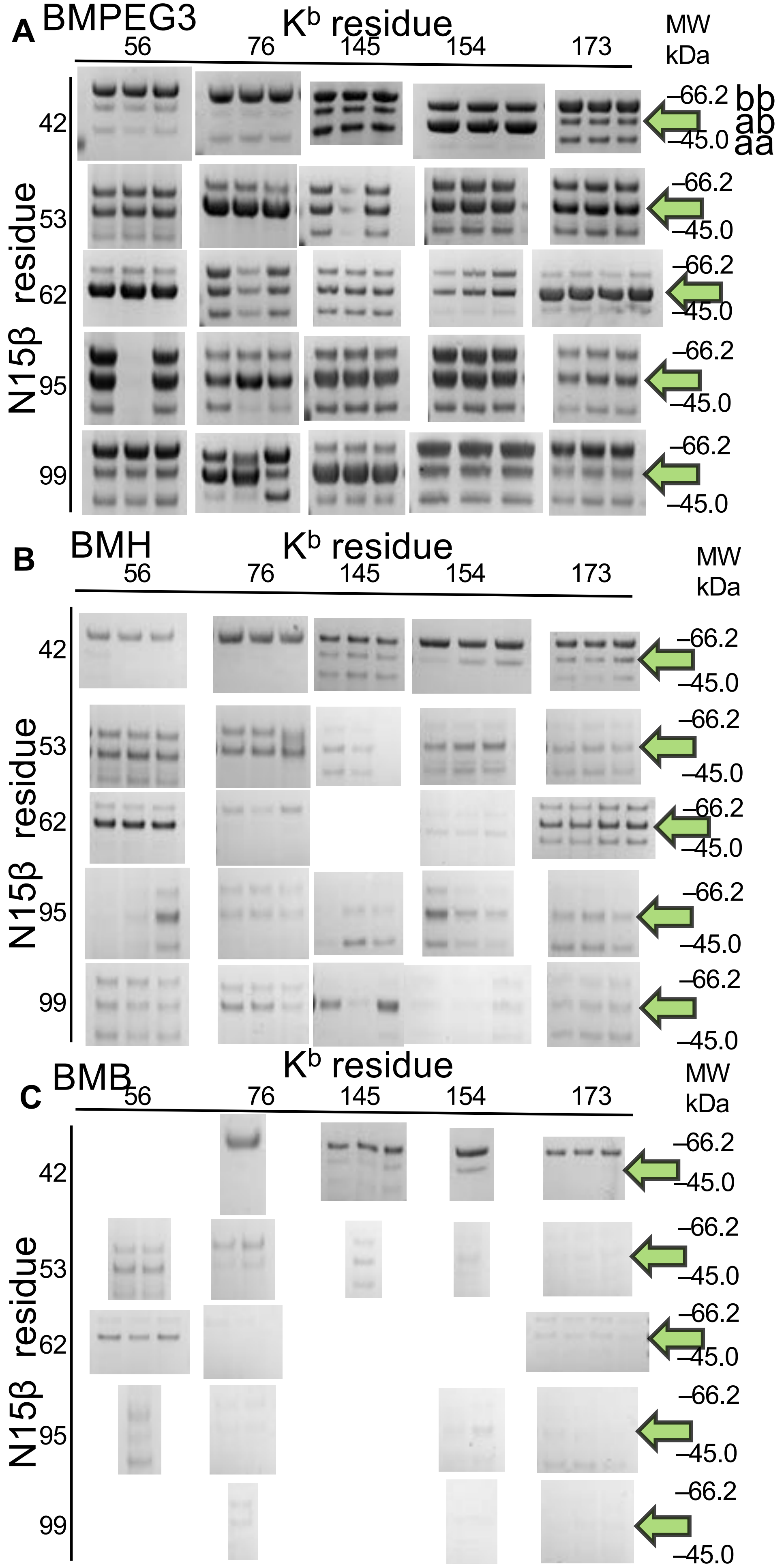
A BMPEG3
K^b residue
56 76 145 154 173
MW kDa
N15β residue
42 53 62 95 99
–66.2 –45.0
bb ab aa
B BMH
K^b residue
56 76 145 154 173
MW kDa
N15β residue
42 53 62 95 99
–66.2 –45.0
C BMB
K^b residue
56 76 145 154 173
MW kDa
N15β residue
42 53 62 95 99
–66.2 –45.0

Figure S2

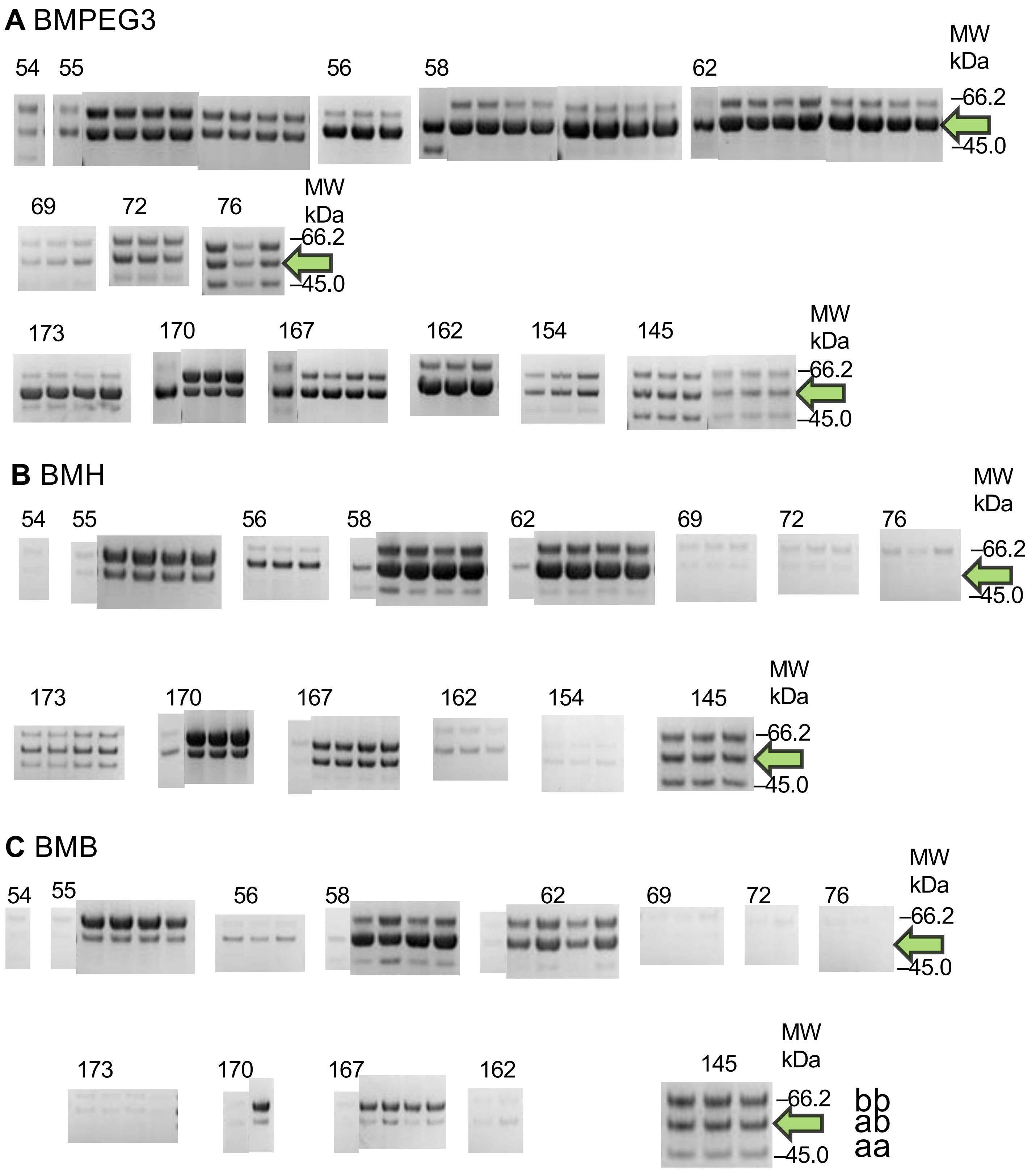
A BMPEG3
54
55
56
58
62
MW kDa
66.2
45.0
69
72
76
MW kDa
66.2
45.0
173
170
167
162
154
145
MW kDa
66.2
45.0
B BMH
54
55
56
58
62
69
72
76
MW kDa
66.2
45.0
173
170
167
162
154
145
MW kDa
66.2
45.0
C BMB
54
55
56
58
62
69
72
76
MW kDa
66.2
45.0
173
170
167
162
145
MW kDa
66.2
45.0
bb
ab
aa

Figure S3

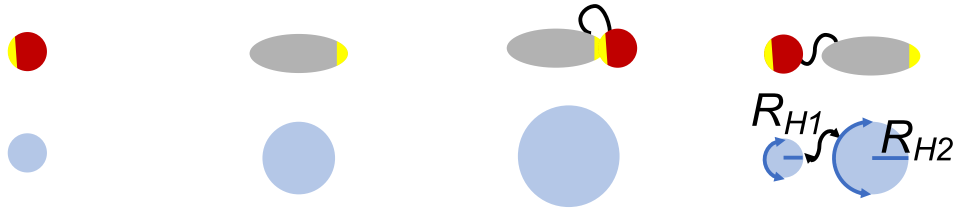

	K^bt2	N15βc1	**homomolecular interaction**	**flexible linkage**
R_H / nm	2.62	2.89	3.49	R_{H1} = 2.62 nm<br>R_{H2} = 2.89 nm
MW / kDa	22.4	27.4	50.2	50.2

Figure S4

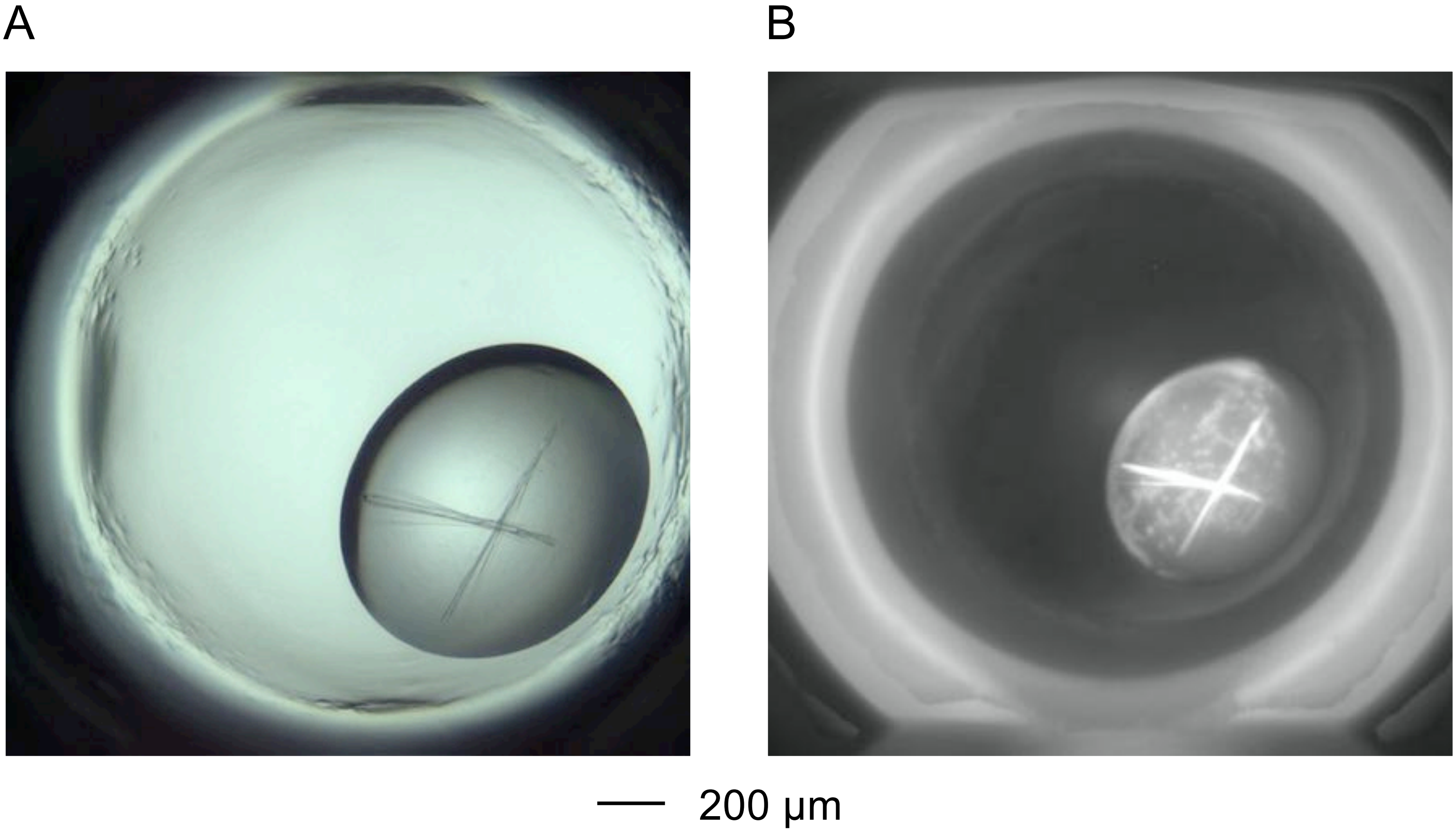

Figure S5

A

SASD / Å		K^{b}-t2 residue				
		56	76	145	154	173
N15β residue	42	39.9	44.7	24.6	10.1	33.5
	53	26.8	24.4	40.1	46.0	39.6
	62	21.0	39.4	49.3	30.1	23.2
	95	49.8	18.4	21.3	35.0	62.8
	99	50.8	14.7	15.1	33.1	63.1

B

Euclidean/ Å		K^{b}-t2 residue				
		56	76	145	154	173
N15β residue	42	34.3	31.9	20.5	9.3	30.2
	53	22.5	21.7	26.3	19.8	30.8
	62	19.9	33.8	32.8	19.7	21.7
	95	34.9	17.7	16.3	17.8	40.2
	99	34.5	14.4	10.6	14.6	38.3

C

Restraints		K^{b}-t2 residue				
		56	76	145	154	173
N15β residue	42				r1	
	53		r2			
	62	r3			r4	r5
	95		r6			
	99		r7	r8		

D

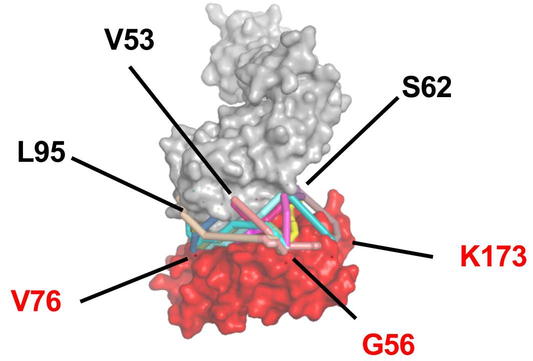

E

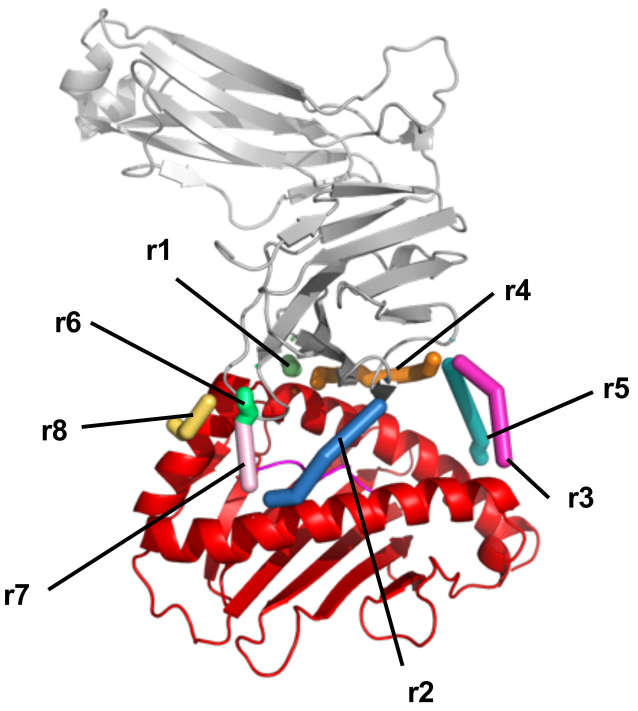

Figure S6

A

K^b-t2	Distance	
Site	SASD	Euclidean
54	30.28	25.19
55	23.46	21.74
56	20.97	19.92
58	21.66	18.94
62	20.38	18.42
69	26.86	24.99
72	33.70	29.71
76	39.40	33.85
145	49.33	32.81
154	30.11	19.69
162	15.45	13.78
167	17.31	15.72
170	18.82	17.43
173	23.19	21.66

B

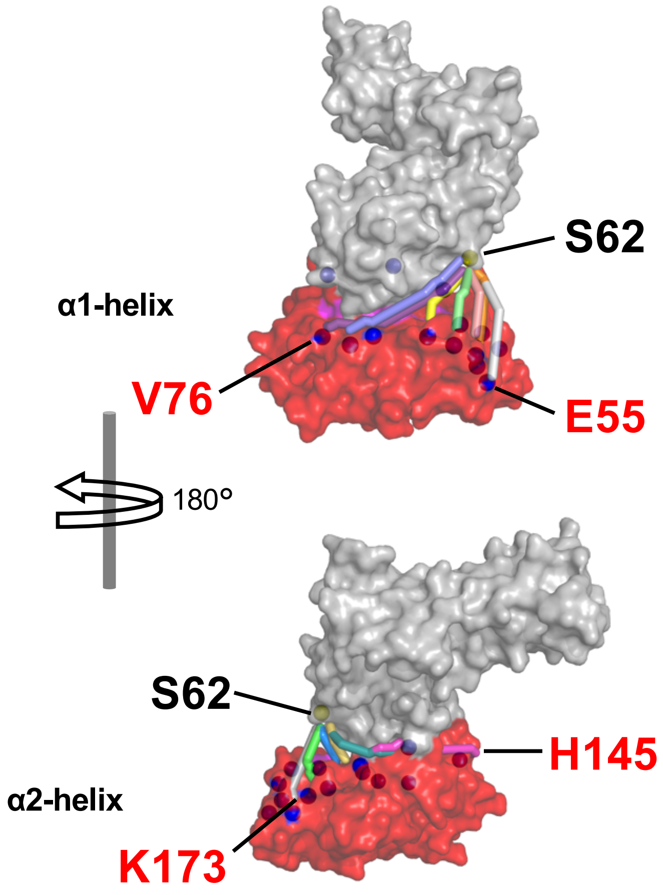

Figure S7

A

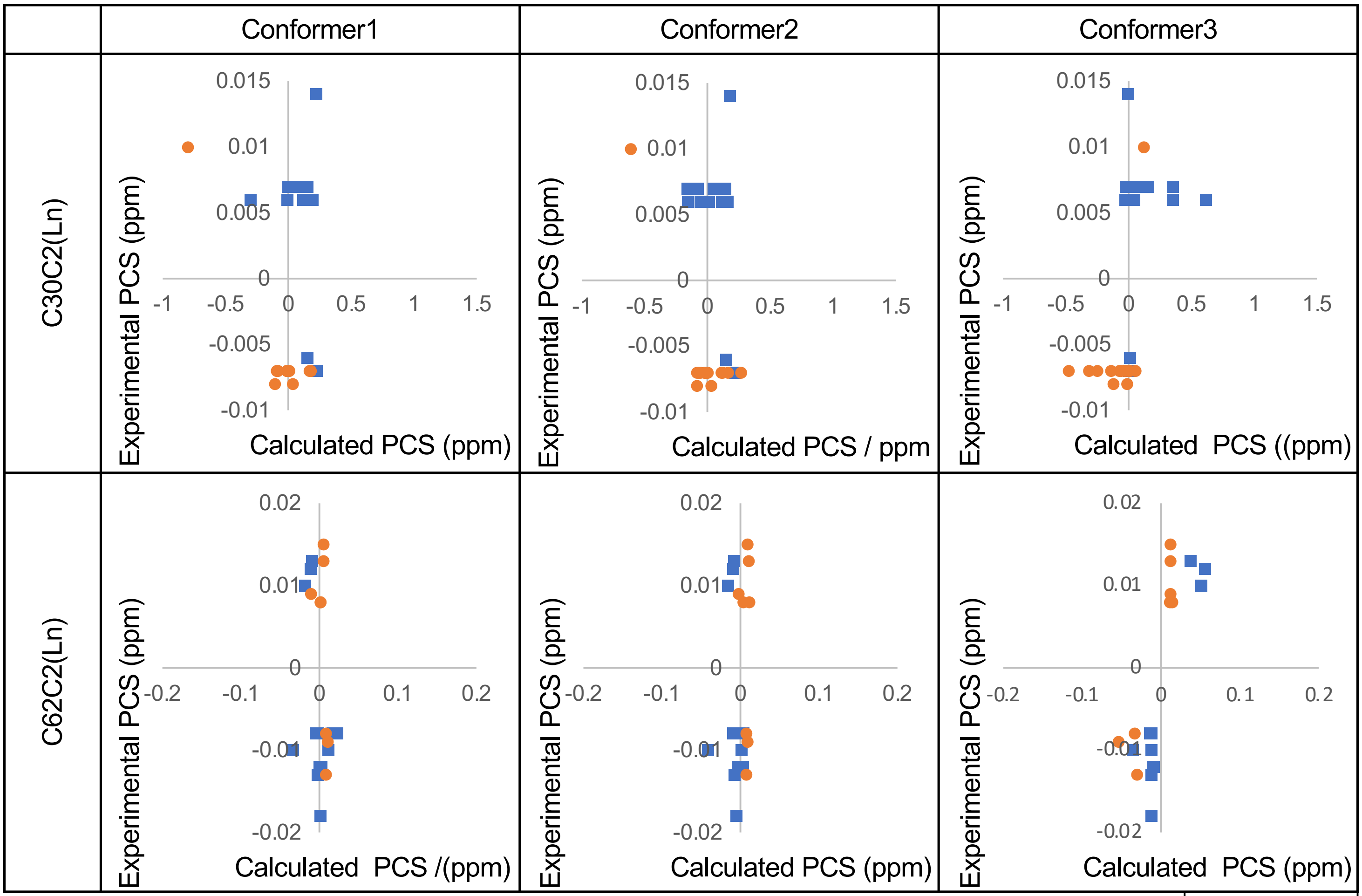

B

Correl.	C30C2(Ln)	C62C2(Ln)
linkage	0.558	0.672
Conformer1	-0.057	-0.252
Conformer2	-0.178	0.148
Conformer3	0.436	0.789

Figure S8

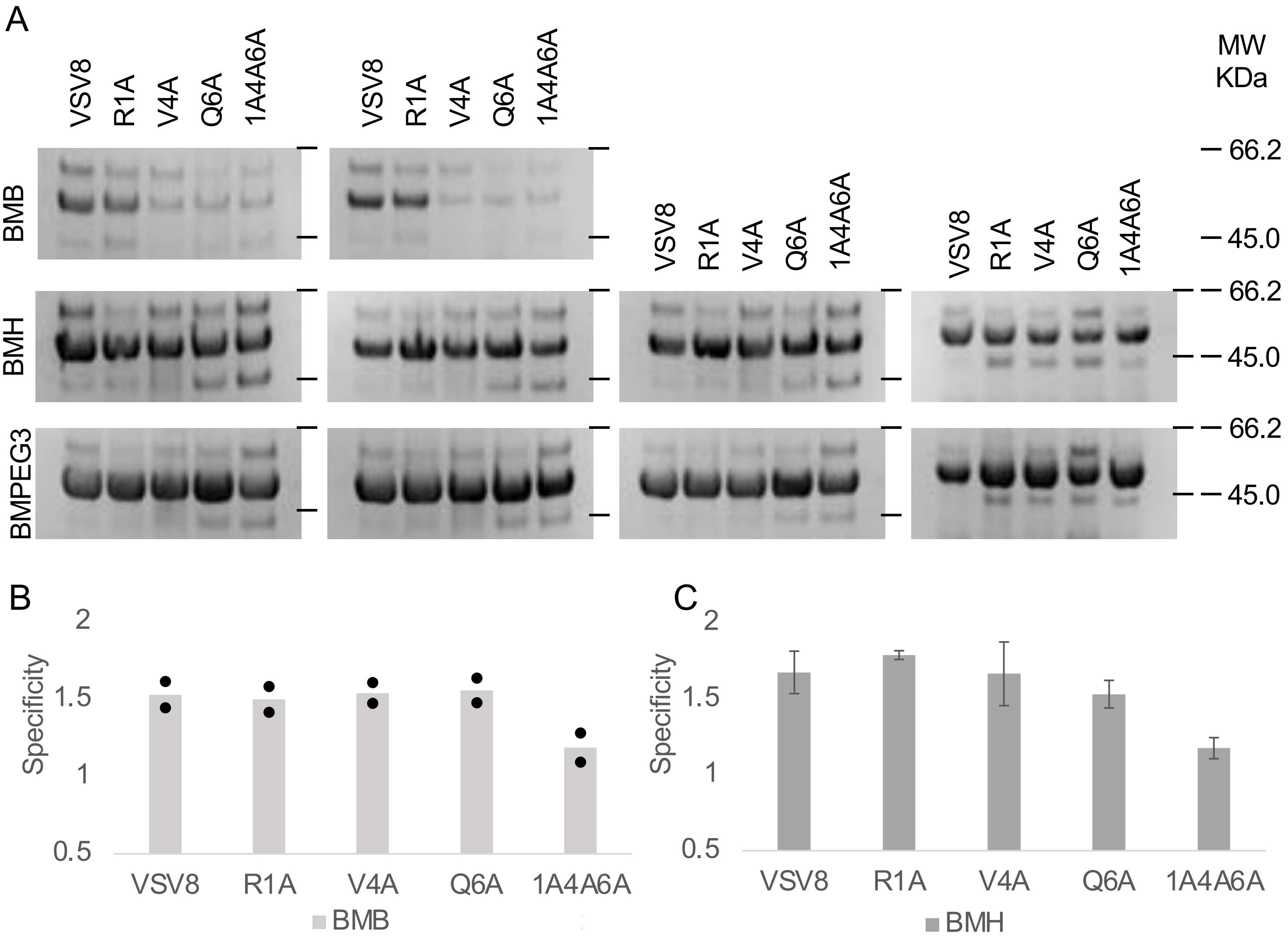
A
VSV8
R1A
V4A
Q6A
1A4A6A
MW KDa
66.2
45.0
BMB
BMH
BMPEG3
B
C
Specificity
2
1.5
1
0.5
BMB
BMH

Figure S9

BMB		K^b residue				
		56	76	145	154	173
N15β residue	42	n.d.	0.00	0.34	0.46	0.05
	53	1.05	0.53	0.43	1.52	0.73
	62	1.88	0.03	n.d.	n.d.	0.98
	95	0.05	0.84	n.d.	1.51	0.03
	99	n.d.	1.00	n.d.	1.59	0.87

Table S1

compound ID	conc.	noise	TCR		MHC		peaks assigned	specificity
	µM		ref. peak int.	S/N	ref. peak int.	S/N		
30-56	187	4500	74888	17	293386	65	254 (406)	0.8
30-68	54	6000	54740	9	176470	29	274 (406)	0.9
30-154	121	5300	131618	25	401319	76	267 (406)	0.8
62-56	106	40	183	5	2379	59	86 (406)	1.8
TCR	200	2600	127042	49	NAN	NAN	179 (227)	NAN
TCR+	200+200	2600	98767	38	NAN	NAN	173 (227)	NAN
MHC	200	600	NAN	NAN	35706	60	139 (179)	NAN
MHC+	200+200	600	NAN	NAN	33771	56	139 (179)	NAN

Table S2

Separate File

Table S3-6

K^b-t2			C30C2(Tb^{3+}) PCS (ppm)		
residue	experimental	linkage	Conformer1	Conformer2	Conformer3
19	0.007	0.03	-0.004	-0.077	0.136
24	0.006	0.308	-0.006	-0.052	0.352
59	0.006	0.271	-0.299	-0.153	0.613
73	0.007	1.461	0.073	-0.158	0.354
79	0.006	0.312	0.117	0.014	0.031
80	0.007	0.305	0.148	0.05	-0.02
101	0.007	0.086	0.135	0.139	0.155
116	0.006	0.114	0.191	0.15	0.042
118	0.007	0.115	0.118	0.081	0.041
123	0.006	0.096	0.146	0.117	0.026
125	0.014	0.051	0.22	0.179	-0.002
128	-0.006	-0.005	0.151	0.15	0.009
130	-0.007	-0.03	0.226	0.221	0.002
135	0.006	0.018	0.162	0.159	-0.028
137	0.006	0.054	0.132	0.118	-0.017
K^b-t2			C30C2(Tm^{3+}) PCS / ppm		
residue	experimental	linkage	Conformer1	Conformer2	Conformer3
3	-0.007	-0.029	-0.012	-0.02	-0.061
4	-0.007	-0.041	-0.011	-0.019	-0.074
48	-0.008	-0.101	0.035	0.034	-0.121
58	-0.007	-0.161	0.169	0.12	-0.249
61	-0.007	-0.256	0.179	0.167	-0.478
65	-0.007	-0.557	0.18	0.268	-1.651
71	-0.007	-0.588	-0.011	0.115	-0.316
76	-0.007	-0.327	-0.094	0.003	0.052
116	-0.008	-0.052	-0.107	-0.084	-0.015
123	-0.007	-0.039	-0.084	-0.069	-0.005
136	-0.007	-0.013	-0.089	-0.082	0.012
152	0.01	0.217	-0.798	-0.607	0.122
166	-0.007	-0.038	0.007	-0.052	-0.14
183	-0.007	-0.023	0.001	-0.002	-0.033
K^b-t2			C62C2(Tb^{3+}) PCS (ppm)		
residue	Experimental	linkage	Conformer1	Conformer2	Conformer3
9	-0.012	-0.008	0	-0.003	-0.01
10	-0.01	-0.012	0.012	0.002	-0.012
23	-0.008	-0.014	0.023	0.004	-0.013
25	-0.018	-0.01	0.002	-0.005	-0.012
26	-0.013	-0.007	-0.002	-0.007	-0.012
34	-0.008	-0.005	-0.004	-0.008	-0.012
35	-0.008	-0.017	-0.001	-0.009	-0.014
37	-0.008	-0.017	0.017	-0.007	-0.013
97	-0.012	-0.008	0.003	0.003	-0.009
109	0.012	0.111	-0.011	-0.009	0.056
110	0.013	0.064	-0.009	-0.008	0.038
151	-0.01	-0.037	-0.033	-0.041	-0.036
161	0.01	0.031	-0.018	-0.016	0.051
K^b-t2			C62C2(Tb^{3+}) PCS / ppm		
residue	experimental	linkage	Conformer1	Conformer2	Conformer3
9	0.015	0.008	0.006	0.009	0.012
11	0.009	0.01	-0.01	-0.002	0.012
24	0.008	0.011	0.002	0.012	0.014
25	0.013	0.007	0.006	0.011	0.012
97	0.008	0.009	0.002	0.004	0.011
109	-0.009	-0.076	0.011	0.009	-0.054
110	-0.008	-0.044	0.009	0.008	-0.033
111	-0.013	-0.031	0.009	0.008	-0.03

Table S7
